# Supplementary material for: Health Risks of Polybrominated Diphenyl Ethers (PBDEs) and Metals at Informal Electronic Waste Recycling Sites
Source: Int J Environ Res Public Health. 2019 Mar 13;16(6):906. doi: 10.3390/ijerph16060906 (PMC6466049; doi:10.3390/ijerph16060906)
Supplement: Supplementary file 1 [file ijerph-16-00906-s001.pdf]

Supplementary information

Materials and Methods for the Polybrominated Diphenyl Ethers (PBDEs) analysis

2.3. Sample collection and preparation

First, the 10ml amber bottles and aluminium foils were treated in the laboratory. The amber bottles were washed with tap water and laboratory detergent, rinsed with a copious amount of tap water, rinsed with distilled water 3 times, treated with acetone and with hexane, and then oven-dried at 120°C for 4 hours to ensure no traces of POPs were present. Aluminium foils (for sample wraps on the field) were treated with acetone and hexane, then oven dried at 120°C to ensure no traces of POPs in the aluminum foil.

On the field, for the soil sampling, each selected site was divided into grids of about 2 m to 10 m wide, depending on the size of the site. Samples were systematically collected from 3 to 6 points within each site. The samples were bulked together for the top soil to form a composite representative sample for the specific site. Soil samples were collected using a soil auger, and a soil trowel was used to the transfer soil from the soil auger into aluminum foil (sample wraps). To avoid cross contamination, the soil probe/auger and trowel were decontaminated (cleaned first with a brush and wiped thoroughly with wipes) before each sample collection at each sampling site. Dust samples were collected using fiber dusting brushes to gently sweep the dust and collect it with a dustpan. The soil and dust samples were wrapped in a treated aluminum foil, labelled, and transported to the laboratory. A total of 71 samples(56 samples from the e-waste recycling sites and 15 samples from control sites) were analysed.The total set consisted of 22 top soil (0-10 cm depth) samples, 30 floor dust samples, 13 roadside dust samples, and 6 direct dust samples. Soil and dust samples were air dried for 7 days, avoiding exposure to sunlight. The samples were homogenized, ground with a mortar and pestle, and sieved through a 1 mm mesh sieve to remove bigger particles. Next, they were transferred into individual 10 ml amber bottles, labelled and stored at -20◦C until shipping to the laboratory for analysis. The samples were collected between May and November 2015.

2.4. Chemicals and materials

All the solvents used for extraction, purification and analysis were of HPLC grade (Spectrum Chemical MFG. Corp., USA). Silica gel (100-200 mesh) and neutral aluminum oxide (100-200 mesh) were for chromatography purpose (Sinopharm Chemical Reagent Co., Ltd, China), and they were activated before use (i.e., first washed with hexane/dichloromethane (v/v, 1/1) and then baked at 180 °C for 2 hours). Acid silica gel (30% w/w) was prepared with activated silica gel and sulphuric acid before use. Anhydrous sodium sulfate (99% purity) and diatomaceous earth (DE, 100% purity) were purchased from Aladdin Ind. Corp, China and Thermo Fisher Scientific respectively. They were baked at 400°C for 4 hours before use to remove any traces of organic matter.

A standard mixture solution of 14 PBDE congeners (BDE-COC) PBDEs (BDE-17, BDE-28, BDE-71, BDE-47, BDE-66, BDE-100, BDE-99, BDE-85, BDE-154, BDE-153, BDE-138, BDE-183, BDE-190, and BDE-209) and Individual standards of 4 PBDEs (BDE-77, BDE-206, BDE-207, BDE-208) and PCB-209 were purchased from Accu Standard, while Isotopically labeled ^{13}C-PCB-208 was purchased from Cambridge Isotope Laboratories. We used ^{13}C-PCB-208 as the surrogate because, we first used chemical ionization source (CI source) to detect PBDEs with the characteristic ionic fragments, and CI source cannot identify the difference between ^{13}C-labeled PBDEs and unlabeled PBDEs. Secondly, ^{13}C-PCB-208 can be identified by CI source, and its characteristic ionic fragments contain ^{13}C labeled carbon. Also, the physiochemical properties of PCBs and PBDEs are similar with PBDEs.

2.5. Sample extraction and cleanup

For the PBDE analysis, from each of the samples, 5g of homogenized sample was thoroughly mixed with 0.6g DE with a mortar and pestle. Each sample was thereafter spiked with 2ng ^{13}C- labeled PCB-208 and 10 ng PCB-209 standards, and allowed a static equilibration of 5 minutes in two cycles. The sample was then extracted using an ASE 350 accelerated solvent extraction system (Dionex, USA) with n-hexane/dichloromethane (v/v, 1/1) at 90◦C, 1500 psi. After extraction, acid washed copper sheets were added to the extracts to remove sulfur present in the samples. The extracts were evaporated to about 10mL under a gentle stream of N_2, and transferred to a conical centrifuge tube. One mL of concentrated sulfuric acid (98%) was added to the concentrated extracts to carbonize part of the impurities present. The supernatants were transferred to a preconditioned glass cleanup column, which was packed with 0.5 cm neutral aluminum oxide, 3.0 cm neutral silica gel, 3.0 cm acid silica gel, and 1.0 cm anhydrous sodium sulfate from the bottom to the top. The columns were then eluted with 20 mL hexane, and the eluent was evaporated to about 0.3 ml and transferred to a 1.5 ml sample vial. After the internal standard (10 ng BDE-77) had been added to the vial, the volume of the solution was made up to 0.5 ml. Many similar previous studies used BDE-77 as internal standard or surrogate [34-35].

2.6. Sample analysis

An Agilent 6890 GC/5975 MSD system operated in negative chemical ion source/ selective ion monitoring (NCI/SIM) mode and equipped with a 15 m DB-XLB column (0.25 mm, 0.1 µm film thickness, J&W) was used for PBDE separation and quantification. The samples (1 µL) were injected in split less mode. Helium was used as carrier gas at a flow rate of 1.2 mL/min, and the temperature program was set as follows: 90 °C for 2min, increased to 320 °C at 15 °C/min and held for 7 min. The temperature of GC inlet, transfer line, ionization source and quadrupole were set at 290°C, 300 °C, and 150 °C. The compounds were monitored at m/z 79 and 81 for 3-7 brominated BDEs, m/z 79, 81, 487 and 489 for BDE-206, 207, 208 and 209, m/z 474, 476 for ^{13}C-PCB-208, and m/z 497.6, 499.6 for PCB-209.

Soil pH was measured using a calibrated pH meter (691, Metrohm AG) in a weight: volume ratio of 1:10 of soil and tap water, adopting the USEPA method 9054D [36]. Total organic content (TOC) of the soil and dust was determined as the weight loss of dried soil (3 hours at 100 ℃) at 550 ℃ for 5 hours [37]. Since PBDEs have a great potential to bind to environmental matrices rich in organic carbon [38], measuring the TOC concentration in the soil and dust samples was used to establish whether there was any correlation between the measured PBDEs and TOC.

2.7. Quality assurance/quality control

A meadow soil collected from a cropland in Liaoning, China (123.90^{0}E, 41.38^{0}N) which was tested and demonstrated to be free of most of the studied PBDEs, was used as matrix blank and matrix spike samples. Twenty ng BDE 206, 207, 208, 209 and 4ng of the other target PBDEs were spiked into 5 g meadow soil to evaluate the method performance. The recoveries for BDE-100, 154, 153, 183, 190, 208, 207, 206 and 209 were 60-107%. For BDE-28, 47, and 99, the recoveries were 32-58%. For all the target compounds, the relative standard deviations of duplicate samples were less than 14 %. The recoveries of BDE-17, 66, 71 and 85 were lower than 20%, therefore they were not excluded from the statistically analysis. For the spiked surrogate ^{13}C-PCB-208 and PCB-209, the average recoveries in all samples were 71% and 84%,

respectively. The method detection limits (MDLs) values characterized as 3 times signal-to-noise ratio were 8 – 164 pg/g for the target PBDEs (supplementary table). The procedural blanks and solvent blanks were analysed simultaneously with samples to check for interferences and contamination. The reported results of PBDEs in the samples were corrected by recoveries of ^{13}C-PCB-208. Three criteria were also used to ensure the correct identification of the target compounds [39]: (a) The GC (gas chromatography) retention times matched those of the authentic standards within ±0.1 min. (b) the signal-to-noise ratio was greater than 3:1; and (c) the isotopic ratios between the quantitative and confirmation ions were within ±15% of the theoretical values. Both ^{13}C-PCB-208 and PCB-209 were used as surrogate to indicate the stability of the recoveries of each sample. In addition, this measure helps to monitor the recoveries of the target compounds at different concentration level.

Materials and Methods for the metals analysis

2.3 Sample collection and preparation

For soil sampling, each site was divided into grids of about 10m radius, and samples were systematically collected from 3 to 6 points within the site. The samples were bulked together for the top soil (0-10 cm depth) to form a composite representative sample for the specific site. Soil samples were collected using a soil auger, and a soil trowel was used in the transfer of soil from the auger into sample wraps. To avoid cross contamination, the soil probe/auger and trowel were decontaminated (cleaned first with a brush and wiped thoroughly with wet wipes) before each sample collection at each sampling site. Dust samples were collected using plastic brushes to gently sweep the dust and collect it with a dustpan. The soil and dust samples were wrapped in an acetone treated aluminum foil, labelled, and transported to the laboratory. Soil and dust samples were air dried for 7 days, homogenized (ground with a mortar and pestle), and sieved through a 1 mm mesh sieve to remove bigger particles, transferred into treated aluminum foil and then into a zip-lock bag, and stored at -20 C. The samples were collected between May and November 2015. A total of 82 samples (62 samples from the e-waste recycling sites and 20 samples from control sites) were analysed.The samples consisted of 29 top soil (0-10 cm depth), 32 floor dust, 16 roadside dust, and 5 direct dust samples from electronics.

2.4 Soil analysis

Soil pH was measured using a calibrated pH meter (691, Metrohm AG) in a (weight: volume) ratio of 1:10 of soil and tap water, adopting the USEPA method 9054D (USEPA 2004). The soil organic matter content (TOC) was determined as the weight loss of dried soil (3hours at 100 ℃) at 550 ℃ for 5 hours(Pansu and Gautheyrou 2006). The total metal content in soil samples was analyzed for trace elements using an X-ray fluorescence (XRF) spectrometer. For the trace metal analyses, about 3g of dry soil was introduced into a sample plastic cup with a 4-μm thick polypropylene film window, with the soil/dust samples settling on the film window. The samples were placed into the XRF spectrometer and analyzed for a fixed period of about 120 s. To check the accuracy of the analysis, each sample was analyzed at

least twice. Acid-purified sand (quartz, SiO_2) was used as the media blank for determining detection limits of major and trace elements and heavy metals.

Supplementary table 1: Median PBDE concentrations (ng/kg) and Exeedance of soil and dusts across various e-waste sites in Lagos

	Top Soil 0-10cm							Floor dust (control soil)				Roadside dust	
**PBDE**	**Control**	**Burning sites**		**Dismantling sites**		**Repair sites**		**Dismantling sites**		**Repair sites**		**Dismantling sites**	
	**Median**	**Median**	**Exceed**	**Median**	**Exceed**	**Median**	**Ex**	**Median**	**Exceed**	**Median**	**Exceed**	**Median**	**Exceed**
**BDE-17**	0± 0.01	3.71		0.08± 13		0.03±0.03		0.13±0.41		0.02±0.03		0.12±0.09	
**BDE-28**	0.005± 0.04	40.54	8108	0.96± 7.6	192	0.02±0.02	4	0.9±4.8	176	0.5±2.5	100	0.18±0.27	36.00
**BDE-71**	0.12± 0.18	433.16	3610	9.4±45	78	0.13±0.13	1.08	23.3±54.2	194	1.6±6.5	13	8.1±3.14	67.50
**BDE-47**	0.04± 0.074	17.05	426	3.67± 41.12	92	0.1±0.1	2.50	6.3±5.2	158	0.54±0.44	14	8.02±5.3	200.50
**BDE-66**	0.02± 0.074	8.42	421	1.49± 22.7	75	0.04±0.04	2.00	1.4±2.8	70	0.14±0.13	7	0.61±0.27	30.50
**BDE-100**	0.06± 0.05	86.02	1434	1.4± 126.3	23	0.31±0.31	5.17	4.1±9	68	0.4±0.15	6.7	4.44±2.54	74.00
**BDE-99**	0.155± 0.172	158.11	1020	10.83± 209.4	70	0.53±0.53	3.42	15±23.8	97	1.8±0.82	12	19±12	122.58
**BDE-85**	0	22.22		0.04± 1.1		0±0		0.21±1.1		0.01±0.02		0.47±0.41	
**BDE-154**	0.055± 0.053	80.25	1460	2.68± 44.5	49	0.21±0.21	3.82	5.4±4.6	98	0.41±0.2	7.45	2.5±1.3	45.45
**BDE-153**	0.085± 0.084	64.19	755	9.93± 159	117	0.24±0.24	2.82	8.9±2.3	105	1.9±6.4	22.35	8.2±3.64	96.47
**BDE-138**	0± 0.015	3.24		0.14± 02.2		0±0		0.82±2.8		0.12±0.16		0.29±0.24	
**BDE-183**	0.065± 0.148	153.73	2365	11.41± 87.4	176	0.2±0.2	3.08	17.6±8.2	270	5±19.1	77	10.76±6	165.54
**BDE-190**	0.06± 0.045	164.12	2735	4.31± 76.2	72	0.19±1.9	3.17	3.3±10.6	55	0.44±0.7	7.33	1.19±0.66	19.83
**BDE-208**	0.12± 0.08	497.38	4145	8.66± 131.8	72	0.1±0.1	0.83	6.33±19.7	53	3±5.6	25	3.28±2.5	27.33
**BDE-207**	0.25± 0.142	952.29	3809	21.03± 291.3	84	0.17±0.17	0.68	18.8±18.6	75	5.7±14.3	23	9.37±7.4	37.48
**BDE-206**	0.145± 0.08	626.79	4323	8.72± 81.4	60	0.07±0.07	0.48	15±11.4	104	2.8±14.2	19	9.49±7.2	65.45
**BDE-209**	3.42± 3	17587	5143	1491±6065.3	436	0.85±0.85	0.25	3009±1176	880	432.74±5850	127	961±1028	280.99
**$\sum_{16}$PBDE**	1.2± 1.2	3311	2759.35	88±1340	73.33	2.34±2.34	1.95	125±166	104.17	25±63	20.83	99±38.5	**82.50**
**$\sum_{17}$PBDE**	4.67± 4	20899	4475.07	1579±7403	338.12	3.19±3.19	0.68	3134±1311	671.092	452±5908	96.79	1060±1055	226.98
**pH**	8.04	7.7		7.89		7.87		8.4		8.31		8.29	
**TOC**	3.13	36.3		7.83		1.51		4.9		3.35		4.43	
Where there s no SD, n=1													

Supplementary table 2: Median metals concentrations (mg/kg) and Exeedance of soil and dusts across various e-waste sites in Lagos

Top Soil 0-10cm								Floor dust (control soil)				Roadside dust	
Metals	Control	Burning sites		Dismantling sites		Repair sites		Dismantling sites		Repair sites		Dismantling sites	
	Median	Median	Exceed	Median	Exceed	Median	Exceed	Median	Exceed	Median	Exceed	Median	Exceed
V	43.8±16	24.2	0.6	50.85±46	1.16	10.2	0.23	38±11	0.87	26±11	0.59	35±13	0.8
Cr	70±19.3	68.7	1.0	103.2±52.2	1.47	49.3	0.70	78±24	1.11	57±15	0.81	62.5±21	0.9
Mn	116.5±27	160.1	1.4	694.9±153.14	5.96	81.4	0.70	680±965	5.84	301±105	2.58	426±73	3.7
Co	1.5±0	1.5	1.0	2.53±2.6	1.69	1.5	1.00	1.5±11	1.00	1.5±0.6	1.00	2.8±1.8	1.9
Ni	18±0.7	194.5	10.8	49.4±116.3	2.74	100	5.56	99.4±88	5.52	28±37	1.56	83±32	4.6
Cu	15±8	12590	839.3	759±5396	50.60	27.6	1.84	766±2904	51.07	82±203	5.47	236±156	15.7
Zn	44±15.3	3152	71.6	810.7±3920	18.43	24.5	0.56	1342±776	30.50	312±286	7.09	924±462	21.0
Ga	9±1.6	1.45	0.2	15.4±8.6	1.71	4	0.44	7.8±2.7	0.87	4±2.7	0.44	5.8±1.7	0.6
Ge	0.25±0.2	0.25	1.0	0.43±0.4	1.72	0.8	3.20	0.6±0.7	2.40	0.25±0.3	1.00	2.1±1.3	8.4
As	0.8±0.4	83.2	104.0	4.93±43	6.16	3.6	4.50	5.7±7	7.13	0.25±1.8	0.31	13±3	16.3
Se	0.25±0	6	24.0	0.2±4.4	0.80	0.25	1.00	0.65±0.6	2.60	0.25±0.38	1.00	0.6±0.07	2.4
Mo	0.5±1.2	8.5	17.0	0.5±10.15	1.00	1.5	3.00	0.5±3	1.00	0.5±0.5	1.00	0.5±0	1.0
Ag	1±2.5	41.1	41.1	5.5±152.2	5.50	1	1.00	34.5±17	34.50	1.3±7.3	1.30	7.7±9.4	7.7
Cd	2.55±0	10.4	4.1	2.55±0.38	1.00	2.55	1.00	2.55±0.18	1.00	2.55±3	1.00	2.55±0	1.0
Sn	1.5±0	2722	1814.7	53.35±1678	35.57	2.8	1.87	349±236	232.67	106±748	70.67	63±44	42.0
Sb	1.5±0.3	1544	1029.3	22.5±1016.5	15.00	1.5	1.00	43±94	28.67	4±13.5	2.67	14±16	9.3
Te	0.5±0	1.5	3.0	1.5±0	3.00	1.5	3.00	1.5±0	3.00	1.5±0.3	3.00	1.5±0	3.0
Ba	78.4±22.5	1378	17.6	256.35±2643	3.27	106.8	1.36	716±400	9.13	227±60	2.90	498±164	6.4
Hg	0.5±0	0.5	1.0	2.45±17	4.90	0.5	1.00	2.3±3.4	4.60	0.5±2.7	1.00	0.18±0.18	0.4
Tl	0.5±0	0.5	1.0	0.6±0.82	1.20	0.5	1.00	0.5±0.3	1.00	0.5±0.18	1.00	0.75±0.2	1.5
Pb	15.7±3	6358	405.0	199±3149	12.68	12.2	0.78	277±264	17.64	57±393	3.63	374±118	23.8
Ta	17.8±2	0.5	0.0	19.9±14.4	1.12	18	1.01	6±6	0.34	16.4±6.4	0.92	21±1	1.2
Fe	11860±2995	7373	0.6	31240±17226	2.63	11000	0.93	32960±77	2.78	16610±668	1.40	24720±702	2.1
Ti	7696±1802	4546	0.6	7804±2968	1.01	5108	0.66	4708±869	0.61	3059±799	0.40	4538±210	0.6
													Where there s no SD, n=1

Supplementary table 3: Median PBDE concentrations (ng/kg) and Exeedance of soil and dusts across various e-waste sites in Ibadan

Top Soil 0-10cm						Floor dust (control soil)		Direct dust			
**PBDE**	**Control**	**Burning sites**		**Dismantling sites**		**Repair sites**		**Dismantling sites**		**Repair sites**	
	**Median**	**Median**	**Exceed**	**Median**	**Exceed**	**Median**	**Exceed**	**Median**	**Exceed**	**Median**	**Exceed**
**BDE-17**	0±0	5.7±6.05		0±0.44		0.095±0.05		0.24±		0.37±1.65	
**BDE-28**	0.03±0.02	18.5±23.1	616.7	0.04±2.5	1.33	1.03±1.03	34.33	1.24	41.33	3.8±12.14	127
**BDE-71**	0.05±0.03	40.14±41	802.8	0.4±106	8	8.78±7.1	176	18.21	364,2	23.2±39	464
**BDE-47**	0.12±0.14	83±105	692.5	0.2±21.3	1,7	4.12±3.4	34,33	64.6	538,33	14.62±38	121.83
**BDE-66**	0.04±0.042	25±32.6	625	0.04±7.04	1	1.1±0.6	27.5	5.4	135	2.53±9.7	63.25
**BDE-100**	0.1±0.08	20±19.3	200	0.17±10.53	1.7	1.51±1.06	15.1	24.2	242	4.8±2.5	48
**BDE-99**	0.27±0.04	107±129.3	396.3	0.42±34.2	1.6	10.64±4.3	39.41	151.2	560	26±27.22	95.2
**BDE-85**	0.005±0.007	8±8.3	1600	0±2.2	0	0.31±0.24	62	4.14	828	1.04±1.2	208
**BDE-154**	0.09±0.06	22.5±19	250	0.1±4	1.1	1.61±2.3	18	12.4	138	4.3±2.14	47.8
**BDE-153**	0.17±0.04	60±59.4	363.6	0.5±14.2	3.03	7.5±21	45.5	33.1	201	12.4±10.35	75.15
**BDE-138**	0±0	3.5±2.4		0±1.7		0,43±0.71		1.73		1.05±0.7	
**BDE-183**	0.07±0.3	38.7±44.4	553	1.2±6.64	17.14	14.5±81	206.57	16.7	238.6	15.5±33.1	221.42
**BDE-190**	0.03±0.014	32.4±23.3	1080	0.1±0.5	3.33	1.5±3.8	50	2.6	87	4.9±3.1	163.33
**BDE-208**	0.15±0.3	173.5±173.5	1157	1.2±4	8	25±21	164.7	14.2	94.5	25.3±176.2	168,33
**BDE-207**	0.27±0.35	367.3±495	1360	1.9±8.2	7.04	78.4±49	290.37	31.7	117.41	42±374	155.6
**BDE-206**	0.19±0.07	120±158.3	632	0.4±6.3	2.11	65±65.45	342.105	31.7	166.8	33.44±599	176
**BDE-209**	9±0.83	5850±7861	650	13±412	1.44	8043±6416	893.7	3181	353.4	1464±68591	162.7
**$\sum_{16}$PBDE**	1,56±0.2	1124±1381	720,51	5,1±228.4	3,269	237±189	151,92	413	264,74	221±1268.2	142
**$\sum_{17}$PBDE**	10,54±1.03	6974±9242	661,67	18,00±639	1,708	8280±6587.5	785,58	3594	340,99	1685±69855	160
**pH**	8.2	8.19		5		8.8		-		9	
**TOC**	3.6	16.78		5.8		5.29		-		18.56	
In case SD is not given, n=1											

Supplementary table 4: Median metal concentrations (mg/kg) and Exeedance of soil and dusts across various e-waste sites in Ibadan

Top Soil 0-10cm						Floor dust (control soil)		Direct dust			
**Metals**	**Control**	**Burning sites**		**Dismantling sites**		**Repair sites**		**Dismantling sites**		**Repair sites**	
	**Median**	**Median**	**Exceed**	**Median**	**Exceed**	**Median**	**Exceed**	**Median**	**Exceed**	**Median**	**Exceed**
**V**	**80**±12	54±3	0.68	55.2±24	0.69	65±21	0.81	922	11.53	111±44	1.39
**Cr**	141±155	123±44	0.87	105±55	0.74	121±25	0.86	14.3	0.10	141±49	1.00
**Mn**	597±16	740±460	1.24	562±805	0.94	758±164	1.27	1607	2.69	797±367	1.34
**Co**	8.3±1.4	1.5±0	0.18	1.5±44	0.18	1.5±2.6	0.18	72.3	8.71	1.5±19	0.18
**Ni**	51.3±17	93±56	1.81	65±62	1.27	43±16	0.84	320	6.24	78±30	1.52
**Cu**	70±27	12749±3976	182.13	1344±4893	19.20	323±351	4.61	42	0.60	321±724	4.59
**Zn**	300±174	3327±901	11.09	1829±3035	6.10	1152±512	3.84	122	0.41	2464±674	8.21
**Ga**	8.6±3	5.2±7	0.60	10±9	1.16	3±2.4	0.35	21	2.44	7.6±4	0.88
**Ge**	1.1±1.2	0.25±0	0.23	0.25±0.33	0.23	0.6±0.4	0.55	1	0.91	0.73±7.3	0.66
**As**	4±5	103±64	25.75	10.3±22	2.58	0.25±2.8	0.06	1.2	0.30	5±30	1.25
**Se**	0.5±0.46	26±30	52.00	4.4±17	8.80	1±2.9	2.00	0.25	0.50	0.9±1.4	1.80
**Mo**	4.2±2.8	8±0.35	1.90	7.2±7.2	1.71	0.6±1.5	0.14	1.3	0.31	2.7±1.4	0.64
**Ag**	14.2±19	16±7	1.13	8.5±28	0.60	5.5±28	0.39	1.35	0.10	15±12	1.06
**Cd**	2.55±0	7±5	2.75	2.55±6	1.00	2.55±1.6	1.00	2.55	1.00	2.7±2.7	1.06
**Sn**	44.4±44	1214±489	27.34	118±160	2.66	970±3948	21.85	1.5	0.03	53±533	1.19
**Sb**	2±0.7	592±346	296.00	52±56	26.00	20±74	10.00	1.5	0.75	48±322	24.00
**Te**	1.5±0	1.5±0	1.00	1.5±0	1.00	1.5±0	1.00	1.5	1.00	1.5±0	1.00
**Ba**	638±269	930±419	1.46	589±250	0.92	469±136	0.74	90	0.14	1049±2502	1.64
**Hg**	1.1±0.8	4.6±5.7	4.18	1.3±7	1.18	0.7±0.95	0.64	0.5	0.45	2.7±1.6	2.45
**Tl**	**0.9**±0.14	10±13	11.11	**0.8**±8	0.89	0.7±0.34	0.78	0.5	0.56	2.25±1.1	2.50
**Pb**	**305**±107	21423±24194	70.24	**2455**±16964	8.05	950±2833	3.11	0.5	0.00	451±752	1.48
**Ta**	**21**±1.3	0.5±0	0.02	14±37	0.67	16±4	0.76	18	0.86	36±32	1.71
**Fe**	35835±6993	31435±7191	0.88	50110±51698	1.40	42540±174	1.19	175000	4.88	57365±2240	1.60
**Ti**	5265±744	6595±2029	1.25	4691±2243	0.89	3478±589	0.66	53190	10.10	4958±1846	0.94
In case SD is not given, n=1											

Supplementary table 5: Median PBDE concentrations (ng/kg) and Exceedance of soil and dusts across various e-waste sites in Aba

**Top Soil 0-10cm (control Roadside dust)**				**Floor dust (control roadside dust)**		**Roadside dust (control roadside dust)**	
**PBDE**	**Control**	**Burning sites**		**Repair sites**		**Repair sites**	
	**Median**	**Median**	**Exceed**	**Median**	**Exceed**	**Median**	**Exceed**
**BDE-17**	0±0.005	0.65±0.9	0	0.03±0.07	0	0.31±0.31	0
**BDE-28**	0.02±0.032	2.3±3	115	0.16±0.6	8	1.44±1.44	72.00
**BDE-71**	0.06±0.136	5.6±7.13	93.33	0.73±2.9	12.17	3.35±3.35	55.83
**BDE-47**	0.47±0.14	5.6±6.2	11.91	1.42±3.53	3.02	3.9±3.9	8.30
**BDE-66**	0.4±0.12	2.3±3.1	5.75	0.2±0.93	0.50	1.6±1.6	4.00
**BDE-100**	0.18±0.13	1.64±1.34	9.11	0.38±0.69	2.11	0.6±0.6	3.33
**BDE-99**	1.08±0.53	11.7±13	10.83	2.55±6.1	2.36	5.84±5.84	5.41
**BDE-85**	0.04±0.017	4.85±6.8	121.25	0.13±0.15	3.25	0	0.00
**BDE-154**	0.13±0.071	7.74±10	59.54	0.43±0.51	3.31	2.46±2.46	18.92
**BDE-153**	0.23±0.071	20.7±27.24	90.00	1.6±2.2	6.96	7.9±7.9	34.35
**BDE-138**	0.02±0.32	1.6±2	80.00	0.1±0.09	5.00	0.54±0.54	27.00
**BDE-183**	0.33±0.5	13.4±17.8	40.61	8.72±6.5	26.42	4.56±4.56	13.82
**BDE-190**	0.07±0.05	7.5±9.3	107.14	0.37±0.46	5.29	2.82±2.82	40.29
**BDE-208**	0.57±0.16	4.4±1.34	7.72	3.4±18.4	5.96	2.57±2.57	4.51
**BDE-207**	1.13±0.35	8.74±9	7.73	10±46	8.85	3.46±3.46	3.06
**BDE-206**	1.2±0.3	3.63±3.2	3.03	6±72.6	5.00	2.15±2.15	1.79
**BDE-209**	71.5±62.8	103.3±67.7	1.44	534±6853	7.47	69±69	0.97
**$\sum_{16}$PBDE**	5.68±1.95	102.25±124	18.00	41.56±140.51	7.32	42±42	7.39
**$\sum_{17}$PBDE**	77.7±61.8	205.55±205.6	2.65	553.15±6982.4	7.12	111.4±11.4	1.43
**pH**		7.52		8.11		8.1	
**TOC**		16.11		2.46		1.5	

Supplementary table 6: Median metals concentrations (mg/kg) and Exceedance of soil and dusts across various e-waste sites in Aba

Top Soil 0-10cm (control soil)						Floor dust (control soil)				Roadside dust (Roadside dustsoil)	
**PBDE**	**Control**	**Burning sites**		**Dismantling sites**		**Dismantling sites**		**Repair sites**		**Dismantling sites**	
	**Median**	**Median**	**Exceed**	**Median**	**Exceed**	**Median**	**Exceed**	**Median**	**Exceed**	**Median**	**Exceed**
**V**	39±44	28±18	0.72	46	1.18	26	0.67	17±8	0.44	42	1.08
**Cr**	63±15	102±48	1.62	197	3.13	188	2.98	42±11	0.67	30	0.48
**Mn**	257±3.5	419±46	1.63	540	2.10	654	2.54	307±45	1.19	274	1.07
**Co**	1.5±0	1.5±0	1.00	1.5	1.00	1.5	1.00	1.5±0	1.00	1.5	1.00
**Ni**	20±6	86±14	4.30	153	7.65	149	7.45	23±10	1.15	16	0.80
**Cu**	21±3	4435±10306	211.19	7880	375.24	13580	646.67	191±476	9.10	23	1.10
**Zn**	85±14	3553±3412	41.80	5650	66.47	5401	63.54	474±243	5.58	119	1.40
**Ga**	10±5	10±7.4	1.00	14.5	1.45	15	1.50	1.8±3	0.18	4.7	0.47
**Ge**	0.5±0.3	0.7±0.4	1.40	0.25	0.50	0.25	0.50	0.25±11	0.50	0.25	0.50
**As**	2±0.3	24±45	12.00	40	20.00	71	35.50	0.25±0.3	0.13	0.25	0.13
**Se**	0.25±0	2.5±4.3	10.00	11.5	46.00	4	16.00	0.25±0.9	1.00	0.25	1.00
**Mo**	0.5±0	14±8	28.00	17	34.00	17.5	35.00	0.5±0.3	1.00	0.5	1.00
**Ag**	1±0	8±5	8.00	14.5	14.50	40	40.00	1±8	1.00	1	1.00
**Cd**	2.55±0	0.5±1.3	0.20	24	9.41	30	11.76	2.55±0	1.00	2.55	1.00
**Sn**	1.5±0	81±1144	54.00	238	158.67	740	493.33	338±1705	225.33	5.5	3.67
**Sb**	1.4±0.4	49±800	35.00	203	145.00	382	272.86	10±22	7.14	1.1	0.79
**Te**	1.5±0	1.5±0	1.00	1.5	1.00	1.5	1.00	1.5±0	1.00	1.5	1.00
**Ba**	60±9	973±5740	16.22	3906	65.10	992	16.53	215±105	3.58	170	2.83
**Hg**	18.5±1	0.5±0.7	0.03	0.5	0.03	0.5	0.03	0.5±0.2	0.03	1	0.05
**Tl**	**0.9**±0.5	0.5±1.9	0.56	**0.5**	0.56	0.5	0.56	0.5±0.15	0.56	0.5	0.56
**Pb**	**0.5**±0	1505±4332	3010.00	**2271**	4542.00	3770	7540.00	150±955	300.00	34	68.00
**Ta**	29±18	0.5±0	0.02	1	0.03	1	0.03	17±8	0.59	26	0.90
**Fe**	21710±9659	51330±7850	2.36	70090	3.23	97260	4.48	18860±7459	0.87	17780	0.82
**Ti**	8314±371	5748±2142	0.69	2608	0.31	4969	0.60	3850±1059	0.46	5488	0.66
In case SD is not given, n=1											

Supplementary table 7: PBDEs:Estimation of Average Daily dose (ADD) via ingestion, inhalation, and dermal uptake via soil and dust at various e-waste in Lagos

	Top Soil 0-10cm									Floor dust (control soil)						Roadside dust		
**PBDE**	**Burning sites**			**Dismantling sites**			**Repair sites**			**Dismantling sites**			**Repair sites**			**Repair sites**		
	**ADD_{ing}**	**ADD_{inh}**	**ADD_{der}**	**ADD_{ing}**	**ADD_{inh}**	**ADD_{der}**	**ADD_{ing}**	**ADD_{inh}**	**ADD_{der}**	**ADD_{ing}**	**ADD_{inh}**	**ADD_{der}**	**ADD_{ing}**	**ADD_{inh}**	**ADD_{der}**	**ADD_{ing}**	**ADD_{inh}**	**ADD_{der}**
**BDE-17**	1.4E-06	6.3E-15	2.0E-03	3.1E-08	1.4E-16	4.3E-05	1.2E-08	5.1E-17	1.6E-05	5.0E-08	2.2E-16	6.9E-05	7.7E-09	3.4E-17	1.1E-05	4.6E-08	2.0E-16	6.4E-05
**BDE-28**	1.6E-05	6.9E-14	2.2E-02	3.7E-07	1.6E-15	5.1E-04	7.7E-09	3.4E-17	1.1E-05	3.4E-07	1.5E-15	4.7E-04	1.9E-07	8.5E-16	2.7E-04	6.9E-08	3.0E-16	9.6E-05
**BDE-71**	1.7E-04	7.3E-13	2.3E-01	3.6E-06	1.6E-14	5.0E-03	5.0E-08	2.2E-16	6.9E-05	8.9E-06	3.9E-14	1.2E-02	6.1E-07	2.7E-15	8.5E-04	3.1E-06	1.4E-14	4.3E-03
**BDE-47**	6.5E-06	2.9E-14	9.1E-03	1.4E-06	6.2E-15	2.0E-03	3.8E-08	1.7E-16	5.3E-05	2.4E-06	1.1E-14	3.4E-03	2.1E-07	9.1E-16	2.9E-04	3.1E-06	1.4E-14	4.3E-03
**BDE-66**	3.2E-06	1.4E-14	4.5E-03	5.7E-07	2.5E-15	7.9E-04	1.5E-08	6.8E-17	2.1E-05	5.4E-07	2.4E-15	7.5E-04	5.4E-08	2.4E-16	7.5E-05	2.3E-07	1.0E-15	3.2E-04
**BDE-100**	3.3E-05	1.5E-13	4.6E-02	5.4E-07	2.4E-15	7.5E-04	1.2E-07	5.3E-16	1.7E-04	1.6E-06	6.9E-15	2.2E-03	1.5E-07	6.8E-16	2.1E-04	1.7E-06	7.5E-15	2.4E-03
**BDE-99**	6.1E-05	2.7E-13	8.4E-02	4.2E-06	1.8E-14	5.8E-03	2.0E-07	9.0E-16	2.8E-04	5.8E-06	2.6E-14	8.0E-03	6.9E-07	3.0E-15	9.6E-04	7.3E-06	3.2E-14	1.0E-02
**BDE-85**	8.5E-06	3.8E-14	1.2E-02	1.5E-08	6.8E-17	2.1E-05	0.0E+00	0.0E+00	0.0E+00	8.1E-08	3.6E-16	1.1E-04	3.8E-09	1.7E-17	5.3E-06	1.8E-07	8.0E-16	2.5E-04
**BDE-154**	3.1E-05	1.4E-13	4.3E-02	1.0E-06	4.5E-15	1.4E-03	8.1E-08	3.6E-16	1.1E-04	2.1E-06	9.2E-15	2.9E-03	1.6E-07	6.9E-16	2.2E-04	9.6E-07	4.2E-15	1.3E-03
**BDE-153**	2.5E-05	1.1E-13	3.4E-02	3.8E-06	1.7E-14	5.3E-03	9.2E-08	4.1E-16	1.3E-04	3.4E-06	1.5E-14	4.7E-03	7.3E-07	3.2E-15	1.0E-03	3.1E-06	1.4E-14	4.4E-03
**BDE-138**	1.2E-06	5.5E-15	1.7E-03	5.4E-08	2.4E-16	7.5E-05	0.0E+00	0.0E+00	0.0E+00	3.1E-07	1.4E-15	4.4E-04	4.6E-08	2.0E-16	6.4E-05	1.1E-07	4.9E-16	1.5E-04
**BDE-183**	5.9E-05	2.6E-13	8.2E-02	4.4E-06	1.9E-14	6.1E-03	7.7E-08	3.4E-16	1.1E-04	6.7E-06	3.0E-14	9.4E-03	1.9E-06	8.5E-15	2.7E-03	4.1E-06	1.8E-14	5.7E-03
**BDE-190**	6.3E-05	2.8E-13	8.7E-02	1.7E-06	7.3E-15	2.3E-03	7.3E-08	3.2E-16	1.0E-04	1.3E-06	5.6E-15	1.8E-03	1.7E-07	7.5E-16	2.3E-04	4.6E-07	2.0E-15	6.3E-04
**BDE-208**	1.9E-04	8.4E-13	2.6E-01	3.3E-06	1.5E-14	4.6E-03	3.8E-08	1.7E-16	5.3E-05	2.4E-06	1.1E-14	3.4E-03	1.2E-06	5.1E-15	1.6E-03	1.3E-06	5.6E-15	1.7E-03
**BDE-207**	3.7E-04	1.6E-12	5.1E-01	8.1E-06	3.6E-14	1.1E-02	6.5E-08	2.9E-16	9.1E-05	7.2E-06	3.2E-14	1.0E-02	2.2E-06	9.7E-15	3.0E-03	3.6E-06	1.6E-14	5.0E-03
**BDE-206**	2.4E-04	1.1E-12	3.3E-01	3.3E-06	1.5E-14	4.6E-03	2.7E-08	1.2E-16	3.7E-05	5.8E-06	2.6E-14	8.0E-03	1.1E-06	4.7E-15	1.5E-03	3.6E-06	1.6E-14	5.1E-03
**BDE-209**	6.8E-03	3.0E-11	9.4E+00	5.7E-04	2.5E-12	7.9E-01	3.3E-07	1.4E-15	4.5E-04	1.2E-03	5.1E-12	1.6E+00	1.7E-04	7.3E-13	2.3E-01	3.7E-04	1.6E-12	5.1E-01
**∑PBDE**	**8.0E-03**	**3.5E-11**	**1.1E+01**	**6.1E-04**	**2.7E-12**	**8.4E-01**	**1.2E-06**	**5.4E-15**	**1.7E-03**	**1.2E-03**	**5.3E-12**	**1.7E+00**	**1.8E-04**	**7.7E-13**	**2.4E-01**	**4.0E-04**	**1.8E-12**	**5.6E-01**

Supplementary table 8.PBDEs: Estimation of Average Daily dose (ADD) via ingestion, inhalation, and dermal uptake via soil and dust at various e-waste sites and in Ibadan

	Top Soil 0-10cm						Floor dust (control soil)			Direct dust					
**PBDE**	**Burning sites**			**Dismantling sites**			**Repair sites**			**Dismantling sites**			**Repair sites**		
	ADD_{ing}	ADD_{inh}	ADD_{der}	ADD_{ing}	ADD_{inh}	ADD_{der}	ADD_{ing}	ADD_{inh}	ADD_{der}	ADD_{ing}	ADD_{inh}	ADD_{der}	ADD_{ing}	ADD_{inh}	ADD_{der}
**BDE-17**	2.2E-06	9.7E-15	3.0E-03	0.0E+00	0.0E+00	0.0E+00	3.6E-08	1.6E-16	5.1E-05	9.2E-08	4.1E-16	1.3E-04	1.4E-07	6.3E-16	2.0E-04
**BDE-28**	7.1E-06	3.1E-14	9.9E-03	1.5E-08	6.8E-17	2.1E-05	4.0E-07	1.7E-15	5.5E-04	4.8E-07	2.1E-15	6.6E-04	1.5E-06	6.4E-15	2.0E-03
**BDE-71**	1.5E-05	6.8E-14	2.1E-02	1.5E-07	6.8E-16	2.1E-04	3.4E-06	1.5E-14	4.7E-03	7.0E-06	3.1E-14	9.7E-03	8.9E-06	3.9E-14	1.2E-02
**BDE-47**	3.2E-05	1.4E-13	4.4E-02	7.7E-08	3.4E-16	1.1E-04	1.6E-06	7.0E-15	2.2E-03	2.5E-05	1.1E-13	3.4E-02	5.6E-06	2.5E-14	7.8E-03
**BDE-66**	9.6E-06	4.2E-14	1.3E-02	1.5E-08	6.8E-17	2.1E-05	4.2E-07	1.9E-15	5.9E-04	2.1E-06	9.1E-15	2.9E-03	9.7E-07	4.3E-15	1.3E-03
**BDE-100**	7.7E-06	3.4E-14	1.1E-02	6.5E-08	2.9E-16	9.1E-05	5.8E-07	2.6E-15	8.0E-04	9.3E-06	4.1E-14	1.3E-02	1.8E-06	8.1E-15	2.6E-03
**BDE-99**	4.1E-05	1.8E-13	5.7E-02	1.6E-07	7.1E-16	2.2E-04	4.1E-06	1.8E-14	5.7E-03	5.8E-05	2.6E-13	8.1E-02	9.9E-06	4.4E-14	1.4E-02
**BDE-85**	3.1E-06	1.4E-14	4.3E-03	0.0E+00	0.0E+00	0.0E+00	1.2E-07	5.3E-16	1.7E-04	1.6E-06	7.0E-15	2.2E-03	4.0E-07	1.8E-15	5.5E-04
**BDE-154**	8.6E-06	3.8E-14	1.2E-02	3.8E-08	1.7E-16	5.3E-05	6.2E-07	2.7E-15	8.6E-04	4.8E-06	2.1E-14	6.6E-03	1.7E-06	7.3E-15	2.3E-03
**BDE-153**	2.3E-05	1.0E-13	3.2E-02	1.9E-07	8.5E-16	2.7E-04	2.9E-06	1.3E-14	4.0E-03	1.3E-05	5.6E-14	1.8E-02	4.8E-06	2.1E-14	6.6E-03
**BDE-138**	1.3E-06	5.9E-15	1.9E-03	0.0E+00	0.0E+00	0.0E+00	1.7E-07	7.3E-16	2.3E-04	6.6E-07	2.9E-15	9.2E-04	4.0E-07	1.8E-15	5.6E-04
**BDE-183**	1.5E-05	6.6E-14	2.1E-02	4.6E-07	2.0E-15	6.4E-04	5.6E-06	2.4E-14	7.7E-03	6.4E-06	2.8E-14	8.9E-03	6.0E-06	2.6E-14	8.3E-03
**BDE-190**	1.2E-05	5.5E-14	1.7E-02	3.8E-08	1.7E-16	5.3E-05	5.8E-07	2.5E-15	8.0E-04	1.0E-06	4.4E-15	1.4E-03	1.9E-06	8.3E-15	2.6E-03
**BDE-208**	6.7E-05	2.9E-13	9.2E-02	4.6E-07	2.0E-15	6.4E-04	9.5E-06	4.2E-14	1.3E-02	5.4E-06	2.4E-14	7.5E-03	9.7E-06	4.3E-14	1.3E-02
**BDE-207**	1.4E-04	6.2E-13	2.0E-01	7.3E-07	3.2E-15	1.0E-03	3.0E-05	1.3E-13	4.2E-02	1.2E-05	5.4E-14	1.7E-02	1.6E-05	7.1E-14	2.2E-02
**BDE-206**	4.6E-05	2.0E-13	6.4E-02	1.5E-07	6.8E-16	2.1E-04	2.5E-05	1.1E-13	3.5E-02	1.2E-05	5.4E-14	1.7E-02	1.3E-05	5.7E-14	1.8E-02
**BDE-209**	2.2E-03	9.9E-12	3.1E+00	5.0E-06	2.2E-14	6.9E-03	3.1E-03	1.4E-11	4.3E+00	1.2E-02	5.4E-11	1.7E+01	5.6E-04	2.5E-12	7.8E-01
**∑PBDE**	**2.7E-03**	**1.2E-11**	**3.7E+00**	**7.6E-06**	**3.3E-14**	**1.0E-02**	**3.2E-03**	**1.4E-11**	**4.4E+00**	**1.2E-02**	**5.5E-11**	**1.7E+01**	**6.4E-04**	**2.8E-12**	**8.9E-01**

Supplementary table 9. PBDEs: Estimation of Average Daily dose (ADD) via ingestion, inhalation, and dermal uptake via soil and dust at various e-waste sites and in Aba

	Top Soil 0-10cm			Floor dust (control soil)			Roadside dust		
**PBDE**	**Burning sites**			**Repair sites**			**Repair sites**		
	**ADD_{ing}**	**ADD_{inh}**	**ADD_{der}**	**ADD_{ing}**	**ADD_{inh}**	**ADD_{der}**	**ADD_{ing}**	**ADD_{inh}**	**ADD_{der}**
**BDE-17**	2.5E-07	1.1E-15	3.5E-04	1.2E-08	5.1E-17	1.6E-05	1.2E-07	5.3E-16	1.7E-04
**BDE-28**	8.8E-07	3.9E-15	1.2E-03	6.1E-08	2.7E-16	8.5E-05	5.5E-07	2.4E-15	7.7E-04
**BDE-71**	2.2E-06	9.5E-15	3.0E-03	2.8E-07	1.2E-15	3.9E-04	1.3E-06	5.7E-15	1.8E-03
**BDE-47**	2.2E-06	9.5E-15	3.0E-03	5.5E-07	2.4E-15	7.6E-04	1.5E-06	6.6E-15	2.1E-03
**BDE-66**	8.8E-07	3.9E-15	1.2E-03	7.7E-08	3.4E-16	1.1E-04	6.1E-07	2.7E-15	8.5E-04
**BDE-100**	6.3E-07	2.8E-15	8.7E-04	1.5E-07	6.4E-16	2.0E-04	2.3E-07	1.0E-15	3.2E-04
**BDE-99**	4.5E-06	2.0E-14	6.2E-03	9.8E-07	4.3E-15	1.4E-03	2.2E-06	9.9E-15	3.1E-03
**BDE-85**	1.9E-06	8.2E-15	2.6E-03	5.0E-08	2.2E-16	6.9E-05	0.0E+00	0.0E+00	0.0E+00
**BDE-154**	3.0E-06	1.3E-14	4.1E-03	1.7E-07	7.3E-16	2.3E-04	9.4E-07	4.2E-15	1.3E-03
**BDE-153**	7.9E-06	3.5E-14	1.1E-02	6.1E-07	2.7E-15	8.5E-04	3.0E-06	1.3E-14	4.2E-03
**BDE-138**	6.1E-07	2.7E-15	8.5E-04	3.8E-08	1.7E-16	5.3E-05	2.1E-07	9.1E-16	2.9E-04
**BDE-183**	5.1E-06	2.3E-14	7.1E-03	3.3E-06	1.5E-14	4.6E-03	1.8E-06	7.7E-15	2.4E-03
**BDE-190**	2.9E-06	1.3E-14	4.0E-03	1.4E-07	6.3E-16	2.0E-04	1.1E-06	4.8E-15	1.5E-03
**BDE-208**	1.7E-06	7.5E-15	2.3E-03	1.3E-06	5.8E-15	1.8E-03	9.9E-07	4.4E-15	1.4E-03
**BDE-207**	3.4E-06	1.5E-14	4.7E-03	3.8E-06	1.7E-14	5.3E-03	1.3E-06	5.9E-15	1.8E-03
**BDE-206**	1.4E-06	6.1E-15	1.9E-03	2.3E-06	1.0E-14	3.2E-03	8.3E-07	3.6E-15	1.1E-03
**BDE-209**	4.0E-05	1.7E-13	5.5E-02	2.1E-04	9.0E-13	2.8E-01	2.6E-05	1.2E-13	3.7E-02
**∑PBDE**	**7.9E-05**	**3.5E-13**	**1.1E-01**	**2.2E-04**	**9.7E-13**	**3.0E-01**	**4.3E-05**	**1.9E-13**	**6.0E-02**

Supplementary table 10. Metal: Estimation of Average Daily dose (ADD) via ingestion, inhalation, and dermal uptake via soil and dust at various e-waste sites and in Lagos

	Top Soil 0-10cm									Floor dust (control soil)						Roadside dust		
**Metals**	**Burning sites**			**Dismantling sites**			**Repair sites**			**Dismantling sites**			**Repair sites**			**Dismantling sites**		
	ADD_{ing}	ADD_{inh}	ADD_{der}	ADD_{ing}	ADD_{inh}	ADD_{der}	ADD_{ing}	ADD_{inh}	ADD_{der}	ADD_{ing}	ADD_{inh}	ADD_{der}	ADD_{ing}	ADD_{inh}	ADD_{der}	ADD_{ing}	ADD_{inh}	ADD_{der}
**V**	9.3E-06	4.1E-14	1.3E-02	2.0E-05	8.6E-14	2.7E-02	3.9E-06	1.7E-14	5.4E-03	1.5E-05	6.4E-14	2.0E-02	1.0E-05	4.4E-14	1.4E-02	1.3E-05	5.9E-14	1.9E-02
**Cr**	2.6E-05	1.2E-13	3.7E-02	4.0E-05	1.7E-13	5.5E-02	1.9E-05	8.4E-14	2.6E-02	3.0E-05	1.3E-13	4.2E-02	2.2E-05	9.7E-14	3.0E-02	2.4E-05	1.1E-13	3.3E-02
**Mn**	6.1E-05	2.7E-13	8.5E-02	2.7E-04	1.2E-12	3.7E-01	3.1E-05	1.4E-13	4.3E-02	2.6E-04	1.2E-12	3.6E-01	1.2E-04	5.1E-13	1.6E-01	1.6E-04	7.2E-13	2.3E-01
**Co**	5.8E-07	2.5E-15	8.0E-04	9.7E-07	4.3E-15	1.3E-03	5.8E-07	2.5E-15	8.0E-04	5.8E-07	2.5E-15	8.0E-04	5.8E-07	2.5E-15	8.0E-04	1.1E-06	4.7E-15	1.5E-03
**Ni**	7.5E-05	3.3E-13	1.0E-01	1.9E-05	8.4E-14	2.6E-02	3.8E-05	1.7E-13	5.3E-02	3.8E-05	1.7E-13	5.3E-02	1.1E-05	4.7E-14	1.5E-02	3.2E-05	1.4E-13	4.4E-02
**Cu**	4.8E-03	2.1E-11	6.7E+00	2.9E-04	1.3E-12	4.0E-01	1.1E-05	4.7E-14	1.5E-02	2.9E-04	1.3E-12	4.1E-01	3.1E-05	1.4E-13	4.4E-02	9.1E-05	4.0E-13	1.3E-01
**Zn**	1.2E-03	5.3E-12	1.7E+00	3.1E-04	1.4E-12	4.3E-01	9.4E-06	4.2E-14	1.3E-02	5.2E-04	2.3E-12	7.1E-01	1.2E-04	5.3E-13	1.7E-01	3.5E-04	1.6E-12	4.9E-01
**Ga**	5.6E-07	2.5E-15	7.7E-04	5.9E-06	2.6E-14	8.2E-03	1.5E-06	6.8E-15	2.1E-03	3.0E-06	1.3E-14	4.2E-03	1.5E-06	6.8E-15	2.1E-03	2.2E-06	9.8E-15	3.1E-03
**Ge**	9.6E-08	4.2E-16	1.3E-04	1.7E-07	7.3E-16	2.3E-04	3.1E-07	1.4E-15	4.3E-04	2.3E-07	1.0E-15	3.2E-04	9.6E-08	4.2E-16	1.3E-04	8.1E-07	3.6E-15	1.1E-03
**As**	3.2E-05	1.4E-13	1.3E-02	1.9E-06	8.4E-15	7.9-04	1.4E-06	6.1E-15	5.8E-04	2.2E-06	9.7E-15	9.1E-04	9.6E-08	4.2E-16	4.0E-05	5.0E-06	2.2E-14	2.1E-03
**Se**	2.3E-06	1.0E-14	3.2E-03	7.7E-08	3.4E-16	1.1E-04	9.6E-08	4.2E-16	1.3E-04	2.5E-07	1.1E-15	3.5E-04	9.6E-08	4.2E-16	1.3E-04	2.3E-07	1.0E-15	3.2E-04
**Mo**	3.3E-06	1.4E-14	4.5E-03	1.9E-07	8.5E-16	2.7E-04	5.8E-07	2.5E-15	8.0E-04	1.9E-07	8.5E-16	2.7E-04	1.9E-07	8.5E-16	2.7E-04	1.9E-07	8.5E-16	2.7E-04
**Ag**	1.6E-05	7.0E-14	2.2E-02	2.1E-06	9.3E-15	2.9E-03	3.8E-07	1.7E-15	5.3E-04	1.3E-05	5.8E-14	1.8E-02	5.0E-07	2.2E-15	6.9E-04	3.0E-06	1.3E-14	4.1E-03
**Cd**	4.0E-06	1.8E-14	5.5E-05	9.8E-07	4.3E-15	1.4E-05	9.8E-07	4.3E-15	1.4E-05	9.8E-07	4.3E-15	1.4E-05	9.8E-07	4.3E-15	1.4E-05	9.8E-07	4.3E-15	1.4E-05
**Sn**	1.0E-03	4.6E-12	1.4E+00	2.0E-05	9.0E-14	2.8E-02	1.1E-06	4.7E-15	1.5E-03	1.3E-04	5.9E-13	1.9E-01	4.1E-05	1.8E-13	5.6E-02	2.4E-05	1.1E-13	3.4E-02
**Sb**	5.9E-04	2.6E-12	8.2E-01	8.6E-06	3.8E-14	1.2E-02	5.8E-07	2.5E-15	8.0E-04	1.7E-05	7.3E-14	2.3E-02	1.5E-06	6.8E-15	2.1E-03	5.4E-06	2.4E-14	7.5E-03
**Te**	5.8E-07	2.5E-15	8.0E-04	5.8E-07	2.5E-15	8.0E-04	5.8E-07	2.5E-15	8.0E-04	5.8E-07	2.5E-15	8.0E-04	5.8E-07	2.5E-15	8.0E-04	5.8E-07	2.5E-15	8.0E-04
**Ba**	5.3E-04	2.3E-12	7.3E-01	9.8E-05	4.3E-13	1.4E-01	4.1E-05	1.8E-13	5.7E-02	2.7E-04	1.2E-12	3.8E-01	8.7E-05	3.8E-13	1.2E-01	1.9E-04	8.4E-13	2.7E-01
**Hg**	1.9E-07	8.5E-16	2.7E-04	9.4E-07	4.2E-15	1.3E-03	1.9E-07	8.5E-16	2.7E-04	8.8E-07	3.9E-15	1.2E-03	1.9E-07	8.5E-16	2.7E-04	6.9E-08	3.0E-16	9.6E-05
**Tl**	1.9E-07	8.5E-16	2.7E-04	2.3E-07	1.0E-15	3.2E-04	1.9E-07	8.5E-16	2.7E-04	1.9E-07	8.5E-16	2.7E-04	1.9E-07	8.5E-16	2.7E-04	2.9E-07	1.3E-15	4.0E-04
**Pb**	2.4E-03	1.1E-11	3.4E+00	7.6E-05	3.4E-13	1.1E-01	4.7E-06	2.1E-14	6.5E-03	1.1E-04	4.7E-13	1.5E-01	2.2E-05	9.7E-14	3.0E-02	1.4E-04	6.3E-13	2.0E-01
**Ta**	1.9E-07	8.5E-16	2.7E-04	7.6E-06	3.4E-14	1.1E-02	6.9E-06	3.0E-14	9.6E-03	2.3E-06	1.0E-14	3.2E-03	6.3E-06	2.8E-14	8.7E-03	8.1E-06	3.6E-14	1.1E-02
**Fe**	2.8E-03	1.2E-11	3.9E+00	1.2E-02	5.3E-11	1.7E+01	4.2E-03	1.9E-11	5.9E+00	1.3E-02	5.6E-11	1.8E+01	6.4E-03	2.8E-11	8.8E+00	9.5E-03	4.2E-11	1.3E+01
**Ti**	1.7E-03	7.7E-12	2.4E+00	3.0E-03	1.3E-11	4.2E+00	2.0E-03	8.7E-12	2.7E+00	1.8E-03	8.0E-12	2.5E+00	1.2E-03	5.2E-12	1.6E+00	1.7E-03	7.7E-12	2.4E+00

Supplementary table 11. Metal: Average Daily dose (ADD) via ingestion, inhalation, and dermal uptake via soil and dust at various e-waste sites and in Ibadan

	Top Soil 0-10cm						Floor dust (control soil)			Direct dust					
**Metals**	**Burning sites**			**Dismantling sites**			**Repair sites**			**Dismantling sites**			**Repair sites**		
	ADD_{ing}	ADD_{inh}	ADD_{der}	ADD_{ing}	ADD_{inh}	ADD_{der}	ADD_{ing}	ADD_{inh}	ADD_{der}	ADD_{ing}	ADD_{inh}	ADD_{der}	ADD_{ing}	ADD_{inh}	ADD_{der}
**V**	2.1E-05	9.1E-14	2.9E-02	2.1E-05	9.4E-14	2.9E-02	2.5E-05	1.1E-13	3.5E-02	3.5E-04	1.6E-12	4.9E-01	4.3E-05	1.9E-13	5.9E-02
**Cr**	4.7E-05	2.1E-13	6.6E-02	4.0E-05	1.8E-13	5.6E-02	4.6E-05	2.0E-13	6.4E-02	5.5E-06	2.4E-14	7.6E-03	5.4E-05	2.4E-13	7.5E-02
**Mn**	2.8E-04	1.3E-12	3.9E-01	2.2E-04	9.5E-13	3.0E-01	2.9E-04	1.3E-12	4.0E-01	6.2E-04	2.7E-12	8.6E-01	3.1E-04	1.4E-12	4.2E-01
**Co**	5.8E-07	2.5E-15	8.0E-04	5.8E-07	2.5E-15	8.0E-04	5.8E-07	2.5E-15	8.0E-04	2.8E-05	1.2E-13	3.9E-02	5.8E-07	2.5E-15	8.0E-04
**Ni**	3.6E-05	1.6E-13	5.0E-02	2.5E-05	1.1E-13	3.5E-02	1.7E-05	7.3E-14	2.3E-02	1.2E-04	5.4E-13	1.7E-01	3.0E-05	1.3E-13	4.2E-02
**Cu**	4.9E-03	2.2E-11	6.8E+00	5.2E-04	2.3E-12	7.2E-01	1.2E-04	5.5E-13	1.7E-01	1.6E-05	7.1E-14	2.2E-02	1.2E-04	5.4E-13	1.7E-01
**Zn**	1.3E-03	5.6E-12	1.8E+00	7.0E-04	3.1E-12	9.7E-01	4.4E-04	2.0E-12	6.1E-01	4.7E-05	2.1E-13	6.5E-02	9.5E-04	4.2E-12	1.3E+00
**Ga**	2.0E-06	8.8E-15	2.8E-03	3.8E-06	1.7E-14	5.3E-03	1.2E-06	5.1E-15	1.6E-03	8.1E-06	3.6E-14	1.1E-02	2.9E-06	1.3E-14	4.0E-03
**Ge**	9.6E-08	4.2E-16	1.3E-04	9.6E-08	4.2E-16	1.3E-04	2.3E-07	1.0E-15	3.2E-04	3.8E-07	1.7E-15	5.3E-04	2.8E-07	1.2E-15	3.9E-04
**As**	4.0E-05	1.7E-13	1.6E-02	4.0E-06	1.7E-14	1.6E-03	9.6E-08	4.2E-16	4.0E-05	4.6E-07	2.0E-15	1.9E-04	1.9E-06	8.5E-15	8.0E-04
**Se**	1.0E-05	4.4E-14	1.4E-02	1.7E-06	7.5E-15	2.3E-03	3.8E-07	1.7E-15	5.3E-04	9.6E-08	4.2E-16	1.3E-04	3.5E-07	1.5E-15	4.8E-04
**Mo**	3.1E-06	1.4E-14	4.3E-03	2.8E-06	1.2E-14	3.8E-03	2.3E-07	1.0E-15	3.2E-04	5.0E-07	2.2E-15	6.9E-04	1.0E-06	4.6E-15	1.4E-03
**Ag**	6.1E-06	2.7E-14	8.5E-03	3.3E-06	1.4E-14	4.5E-03	2.1E-06	9.3E-15	2.9E-03	5.2E-07	2.3E-15	7.2E-04	5.8E-06	2.5E-14	8.0E-03
**Cd**	2.7E-06	1.2E-14	3.7E-05	9.8E-07	4.3E-15	1.4E-05	9.8E-07	4.3E-15	1.4E-05	9.8E-07	4.3E-15	1.4E-05	1.0E-06	4.6E-15	1.4E-05
**Sn**	4.7E-04	2.1E-12	6.5E-01	4.5E-05	2.0E-13	6.3E-02	3.7E-04	1.6E-12	5.2E-01	5.8E-07	2.5E-15	8.0E-04	2.0E-05	9.0E-14	2.8E-02
**Sb**	2.3E-04	1.0E-12	3.2E-01	2.0E-05	8.8E-14	2.8E-02	7.7E-06	3.4E-14	1.1E-02	5.8E-07	2.5E-15	8.0E-04	1.8E-05	8.1E-14	2.6E-02
**Te**	5.8E-07	2.5E-15	8.0E-04	5.8E-07	2.5E-15	8.0E-04	5.8E-07	2.5E-15	8.0E-04	5.8E-07	2.5E-15	8.0E-04	5.8E-07	2.5E-15	8.0E-04
**Ba**	3.6E-04	1.6E-12	5.0E-01	2.3E-04	1.0E-12	3.1E-01	1.8E-04	7.9E-13	2.5E-01	3.5E-05	1.5E-13	4.8E-02	4.0E-04	1.8E-12	5.6E-01
**Hg**	1.8E-06	7.8E-15	2.4E-03	5.0E-07	2.2E-15	6.9E-04	2.7E-07	1.2E-15	3.7E-04	1.9E-07	8.5E-16	2.7E-04	1.0E-06	4.6E-15	1.4E-03
**Tl**	3.8E-06	1.7E-14	5.3E-03	3.1E-07	1.4E-15	4.3E-04	2.7E-07	1.2E-15	3.7E-04	1.9E-07	8.5E-16	2.7E-04	8.6E-07	3.8E-15	1.2E-03
**Pb**	8.2E-03	3.6E-11	1.1E+01	9.4E-04	4.2E-12	1.3E+00	3.6E-04	1.6E-12	5.1E-01	1.9E-07	8.5E-16	2.7E-04	1.7E-04	7.6E-13	2.4E-01
**Ta**	1.9E-07	8.5E-16	2.7E-04	5.4E-06	2.4E-14	7.5E-03	6.1E-06	2.7E-14	8.5E-03	6.9E-06	3.0E-14	9.6E-03	1.4E-05	6.1E-14	1.9E-02
**Fe**	1.2E-02	5.3E-11	1.7E+01	1.9E-02	8.5E-11	2.7E+01	1.6E-02	7.2E-11	2.3E+01	6.7E-02	3.0E-10	9.3E+01	2.2E-02	9.7E-11	3.1E+01
**Ti**	2.5E-03	1.1E-11	3.5E+00	1.8E-03	7.9E-12	2.5E+00	1.3E-03	5.9E-12	1.9E+00	2.0E-02	9.0E-11	2.8E+01	1.9E-03	8.4E-12	2.6E+00

Supplementary table 12. Metal: Average Daily dose (ADD) via ingestion, inhalation, and dermal uptake via soil and dust at various e-waste sites and in Aba

	Top Soil 0-10cm						Floor dust						Roadside dust		
**Metals**	**Burning sites**			**Dismantling sites**			**Dismantling sites**			**Repair sites**					
	ADD_{ing}	ADD_{inh}	ADD_{der}	ADD_{ing}	ADD_{inh}	ADD_{der}	ADD_{ing}	ADD_{inh}	ADD_{der}	ADD_{ing}	ADD_{inh}	ADD_{der}	ADD_{ing}	ADD_{inh}	ADD_{der}
**V**	1.1E-05	4.7E-14	1.5E-02	1.8E-05	7.8E-14	2.4E-02	1.0E-05	4.4E-14	1.4E-02	6.5E-06	2.9E-14	9.1E-03	1.6E-05	7.1E-14	2.2E-02
**Cr**	3.9E-05	1.7E-13	5.4E-02	7.6E-05	3.3E-13	1.0E-01	7.2E-05	3.2E-13	1.0E-01	1.6E-05	7.1E-14	2.2E-02	1.2E-05	5.1E-14	1.6E-02
**Mn**	1.6E-04	7.1E-13	2.2E-01	2.1E-04	9.1E-13	2.9E-01	2.5E-04	1.1E-12	3.5E-01	1.2E-04	5.2E-13	1.6E-01	1.1E-04	4.6E-13	1.5E-01
**Co**	5.8E-07	2.5E-15	8.0E-04	5.8E-07	2.5E-15	8.0E-04	5.8E-07	2.5E-15	8.0E-04	5.8E-07	2.5E-15	8.0E-04	5.8E-07	2.5E-15	8.0E-04
**Ni**	3.3E-05	1.5E-13	4.6E-02	5.9E-05	2.6E-13	8.1E-02	5.7E-05	2.5E-13	7.9E-02	8.8E-06	3.9E-14	1.2E-02	6.1E-06	2.7E-14	8.5E-03
**Cu**	1.7E-03	7.5E-12	2.4E+00	3.0E-03	1.3E-11	4.2E+00	5.2E-03	2.3E-11	7.2E+00	7.3E-05	3.2E-13	1.0E-01	8.8E-06	3.9E-14	1.2E-02
**Zn**	1.4E-03	6.0E-12	1.9E+00	2.2E-03	9.6E-12	3.0E+00	2.1E-03	9.1E-12	2.9E+00	1.8E-04	8.0E-13	2.5E-01	4.6E-05	2.0E-13	6.3E-02
**Ga**	3.8E-06	1.7E-14	5.3E-03	5.6E-06	2.5E-14	7.7E-03	5.8E-06	2.5E-14	8.0E-03	6.9E-07	3.0E-15	9.6E-04	1.8E-06	8.0E-15	2.5E-03
**Ge**	2.7E-07	1.2E-15	3.7E-04	9.6E-08	4.2E-16	1.3E-04	9.6E-08	4.2E-16	1.3E-04	9.6E-08	4.2E-16	1.3E-04	9.6E-08	4.2E-16	1.3E-04
**As**	9.2E-06	4.1E-14	3.8E-03	1.5E-05	6.8E-14	6.4E-03	2.7E-05	1.2E-13	1.1E-02	9.6E-08	4.2E-16	4.0E-05	9.6E-08	4.2E-16	4.0E-05
**Se**	9.6E-07	4.2E-15	1.3E-03	4.4E-06	1.9E-14	6.1E-03	1.5E-06	6.8E-15	2.1E-03	9.6E-08	4.2E-16	1.3E-04	9.6E-08	4.2E-16	1.3E-04
**Mo**	5.4E-06	2.4E-14	7.5E-03	6.5E-06	2.9E-14	9.1E-03	6.7E-06	3.0E-14	9.3E-03	1.9E-07	8.5E-16	2.7E-04	1.9E-07	8.5E-16	2.7E-04
**Ag**	3.1E-06	1.4E-14	4.3E-03	5.6E-06	2.5E-14	7.7E-03	1.5E-05	6.8E-14	2.1E-02	3.8E-07	1.7E-15	5.3E-04	3.8E-07	1.7E-15	5.3E-04
**Cd**	1.9E-07	8.5E-16	2.7E-04	9.2E-06	4.1E-14	1.3E-02	1.2E-05	5.1E-14	1.6E-02	9.8E-07	4.3E-15	1.4E-03	9.8E-07	4.3E-15	1.4E-03
**Sn**	3.1E-05	1.4E-13	4.3E-02	9.1E-05	4.0E-13	1.3E-01	2.8E-04	1.3E-12	3.9E-01	1.3E-04	5.7E-13	1.8E-01	2.1E-06	9.3E-15	2.9E-03
**Sb**	1.9E-05	8.3E-14	2.6E-02	7.8E-05	3.4E-13	1.1E-01	1.5E-04	6.5E-13	2.0E-01	3.8E-06	1.7E-14	5.3E-03	4.2E-07	1.9E-15	5.9E-04
**Te**	5.8E-07	2.5E-15	8.0E-04	5.8E-07	2.5E-15	8.0E-04	5.8E-07	2.5E-15	8.0E-04	5.8E-07	2.5E-15	8.0E-04	5.8E-07	2.5E-15	8.0E-04
**Ba**	3.7E-04	1.6E-12	5.2E-01	1.5E-03	6.6E-12	2.1E+00	3.8E-04	1.7E-12	5.3E-01	8.3E-05	3.6E-13	1.1E-01	6.5E-05	2.9E-13	9.1E-02
**Hg**	1.9E-07	8.5E-16	2.7E-04	1.9E-07	8.5E-16	2.7E-04	1.9E-07	8.5E-16	2.7E-04	1.9E-07	8.5E-16	2.7E-04	3.8E-07	1.7E-15	5.3E-04
**Tl**	1.9E-07	8.5E-16	2.7E-04	1.9E-07	8.5E-16	2.7E-04	1.9E-07	8.5E-16	2.7E-04	1.9E-07	8.5E-16	2.7E-04	1.9E-07	8.5E-16	2.7E-04
**Pb**	5.8E-04	2.5E-12	8.0E-01	8.7E-04	3.8E-12	1.2E+00	1.4E-03	6.4E-12	2.0E+00	5.8E-05	2.5E-13	8.0E-02	1.3E-05	5.8E-14	1.8E-02
**Ta**	1.9E-07	8.5E-16	2.7E-04	3.8E-07	1.7E-15	5.3E-04	3.8E-07	1.7E-15	5.3E-04	6.5E-06	2.9E-14	9.1E-03	1.0E-05	4.4E-14	1.4E-02
**Fe**	2.0E-02	8.7E-11	2.7E+01	2.7E-02	1.2E-10	3.7E+01	3.7E-02	1.6E-10	5.2E+01	7.2E-03	3.2E-11	1.0E+01	6.8E-03	3.0E-11	9.5E+00
**Ti**	2.2E-03	9.7E-12	3.1E+00	1.0E-03	4.4E-12	1.4E+00	1.9E-03	8.4E-12	2.6E+00	1.5E-03	6.5E-12	2.1E+00	2.1E-03	9.3E-12	2.9E+00

Supplementary table 13: Hazard Quotient (HQ) via ingestion, inhalation, and dermal uptake via soil and dust at various e-waste sites and in Lagos

PBDE	RfD	Top Soil 0-10cm									Floor dust (control soil)						Roadside dust		
		Burning sites			Dismantling sites			Repair sites			Dismantling sites			Repair sites			Dismantling sites		
		HQi_{ng}	HQ_{inh}	HQ_{der}	HQ_{ing}	HQ_{inh}	HQ_{der}	HQ_{ing}	HQ_{inh}	HQ_{der}	HQ_{ing}	HQ_{inh}	HQ_{der}	HQ_{ing}	HQ_{inh}	HQ_{der}	HQ_{ing}	HQ_{inh}	HQ_{der}
**BDE-47**	1.00E-04	6.5E-05		9.1E-02	1.41E-05		2.0E-02	3.8E-07		5.3E-04	2.42E-05		3.36E-02	2.07E-06		2.88E-03	3.08E-05		4.27E-02
**BDE-99**	1.00E-04	6.1E-04		8.4E-01	4.16E-05		5.8E-02	2.0E-06		2.8E-03	5.80E-05		8.04E-02	6.91E-06		9.59E-03	7.30E-05		1.01E-01
**BDE-153**	2.00E-04	1.2E-04		1.7E-01	1.91E-05		2.6E-02	4.6E-01		6.4E-04	1.71E-05		2.37E-02	3.65E-06		5.06E-03	1.57E-05		2.18E-02
**BDE-209**	7.00E-03	9.6E-04		1.3E+00	8.18E-05		1.1E-01	4.7E-07		6.5E-05	1.65E-04		2.29E-01	2.37E-05		3.29E-02	5.27E-05		7.31E-02
**∑HQ=HI**		**1.76E**-03		**2.4E+00**	**1.57E**-04		**2.2E**-01	**2.93E**-06		**4.06E**-03	**2.64E**-04		**3.67E**-01	**3.64E**-05		**5.04E-02**	**1.72E-04**		**2.39E**-01

Supplementary table 14: Hazard Quotient (HQ) via ingestion, inhalation, dermal uptake via soil and dust at various e-waste sites and in Ibadan

PBDE	RfD	Top Soil 0-10cm						Floor dust (control soil)			Direct dust					
		Burning sites			Dismantling sites			Repair sites			Dismantling sites			Repair sites		
		HQ_{ing}	HQ_{inh}	HQ_{der}	HQ_{ing}	HQ_{inh}	HQ_{der}	HQ_{ing}	HQ_{inh}	HQ_{der}	HQ_{ing}	HQ_{inh}	HQ_{der}	HQ_{ing}	HQ_{inh}	HQ_{der}
**BDE-47**	1.00E-04	3.2E-01		4.4+02	7.7E-04		1.1E+00	1.6E-02		2.2E+01	2.48E-01		3.4E+02	5.61E-02		7.8E+01
**BDE-99**	1.00E-04	4.1E-01		5.7E+02	1.6E-03		2.2E+00	4.1E-02		5.7E+01	5.81E-01		8.1E+02	9.87E-02		1.4E+02
**BDE-153**	2.00E-04	1.2E-01		1.6E+02	9.6E-04		1.3E+00	1.4E-02		2.0E+01	6.35E-02		8.8E+01	2.38E-02		3.3E+01
**BDE-209**	7.00E-03	3.2E-01		4.5E-02	7.1E-04		9.9E-01	4.4E-01		6.1E+02	1.74E+00		2.4E+03	8.03E-02		1.1E+02
**∑HQ=HI**		**1.2E+00**		**1.6E+03**	**4.1E-03**		**5.6E+00**	**5.1E-01**		**7.1E+02**	**2.64E+00**		**3.7E+03**	**2.59E-01**		**3.6E+02**

Supplementary table 15: Hazard Quotient (HQ) via ingestion, inhalation, dermal uptake via soil and dust at various e-waste sites and in Aba

		Top Soil 0-10cm			Floor dust (control soil)			Roadside dust		
**PBDE**	**RfD**	**Burning sites**			**Repair sites**			**Repair sites**		
		HQ_{ing}	R_{ing}	R_{der}	HQ_{ing}	R_{ing}	R_{der}	HQ_{ing}	R_{ing}	R_{der}
**BDE-47**	1.00E-04	2.2E-02		3.0E+01	5.45E-03		7.6E+00	1.50E-02		2.1E+01
**BDE-99**	1.00E-04	4.5E-02		6.2E+01	9.79E-03		1.4E+01	2.24E-02		3.1E+01
**BDE-153**	2.00E-04	4.0E-02		5.5E+01	3.07E-03		4.3E+00	1.52E-02		2.1E+01
**BDE-209**	7.00E-03	5.7E-03		7.9E+00	2.93E-02		4.1E+01	3.78E-03		5.2E+00
**∑HQ=HI**		**1.1E-01**		**1.6E+02**	**4.76E-02**		**6.6E+01**	**5.64E-02**		7.8E+01

Supplementary table 16: Total HI Estimate for PBDEs and Metals for non-cancer Effects (log transformed data)

Samples	Location	Activity	Ingestion	Inhalation	Dermal contact
Direct dust	Ibadan	Dismantling	-0.05	-6	3.73
		Repair	0.18	-6.89	3.6
Floor dust	Lagos	Dismantling	-0.48	-6.92	3.23
		Repair	-0.18	-7.17	3.18
	Ibadan	Repair	-0.16	-7.02	3.36
	Aba	Repair	-0.13	-6.85	3.74
		Dismantling	-0.16	-7.09	3.15
Roadside Dust	Lagos	Dismantling	-0.06	-6.96	3.3
	Aba	Repair	0	-6.96	3.26
Soil	Lagos	Burning	0.26	-6.92	4.18
		Dismantling	-0.08	-6.77	3.36
		Repair	-0.14	-7	3.18
	Ibadan	Burning	-0.05	-6.77	3.83
		Dismantling	-0.17	-6.96	3.38
	Aba	Burning	-0.7	-6.89	3.2
		Dismantling	-0.34	-6.96	3.6
Safe Limit			0	0	0

Supplementary table 17: Hazard Quotient (HQ) ingestion, inhalation, dermal in soil and dust at various e-waste sites and in Lagos

Metals	Top Soil 0-10cm									Floor dust (control soil)						Roadside dust		
	Burning sites			Dismantling sites			Repair sites			Dismantling sites			Repair sites			Dismantling sites		
	HQ_{ing}	HQ_{inh}	HQ_{der}	HQ_{ing}	HQ_{inh}	HQ_{der}	HQ_{ing}	HQ_{inh}	HQ_{der}	HQ_{ing}	HQ_{inh}	HQ_{der}	HQ_{ing}	HQ_{inh}	HQ_{der}	HQ_{ing}	HQ_{inh}	HQ_{der}
**V**	1.9E-03	4.1E-10	9.91E+01	3.9E-03	8.6E-10	2.08E+02	7.8E-04	1.7E-10	4.18E+01	2.9E-03	6.4E-10	1.56E+02	2.0E-03	4.4E-10	1.07E+02	2.7E-03	5.9E-10	1.4E+02
**Cr**	8.8E-03	1.2E-09	4.88E+02	1.3E-02	1.7E-09	7.33E+02	6.3E-03	8.4E-10	3.50E+02	1.0E-02	1.3E-09	5.54E+02	7.3E-03	9.7E-10	4.05E+02	8.0E-03	1.1E-09	4.4E+02
**Mn**	4.4E-04	5.4E-09	6.09E-01	1.9E-03	2.4E-08	2.64E+00	2.2E-04	2.8E-09	3.10E-01	1.9E-03	2.3E-08	2.59E+00	8.3E-04	1.0E-08	1.15E+00	1.2E-03	1.4E-08	1.6E+00
**Co**	1.9E-04	4.2E-10	2.66E-01	3.2E-04	7.1E-10	4.49E-01	1.9E-04	4.2E-10	2.66E-01	1.9E-04	4.2E-10	2.66E-01	1.9E-04	4.2E-10	2.66E-01	3.6E-04	7.9E-10	5.0E-01
**Ni**	6.8E-03	2.4E-08	2.35E+02	1.7E-03	6.0E-09	5.98E+01	3.5E-03	1.2E-08	1.21E+02	3.5E-03	1.2E-08	1.20E+02	9.8E-04	3.4E-09	3.39E+01	2.9E-03	1.0E-08	1.0E+02
**Cu**	1.2E-01	1.5E-20	1.68E+02	7.3E-03	9.2E-22	1.01E+01	2.6E-04	3.3E-23	3.67E-01	7.4E-03	9.3E-22	1.02E+01	7.9E-04	9.9E-23	1.09E+00	2.3E-03	2.9E-22	3.1E+00
**Zn**	4.0E-03		5.60E+00	1.0E-03		1.44E+00	3.1E-05		4.35E-02	1.7E-03		2.38E+00	4.0E-04		5.54E-01	1.2E-03		1.6E+00
**As**	1.1E-01	9.4E-09	1.43E+01	6.3E-03	5.6E-10	2.63E+00	4.6E-03	4.1E-10	1.92E+00	7.3E-03	6.4E-10	3.0E+00	3.2E-04	2.8E-11	1.33E-01	1.7E-02	1.5E-09	6.9E+00
**Se**	4.6E-04	5.1E-13	6.39E-01	1.5E-05	1.7E-14	2.13E-02	1.9E-05	2.1E-14	2.66E-02	5.0E-05	5.5E-14	6.92E-02	1.9E-05	2.1E-14	2.66E-02	4.6E-05	5.1E-14	6.4E-02
**Mo**	6.5E-04		9.05E-01	3.8E-05		5.33E-02	1.2E-04		1.60E-01	3.8E-05		5.33E-02	3.8E-05		5.33E-02	3.8E-05		5.3E-02
**Ag**	3.2E-03		1.09E+02	4.2E-04		1.46E+01	7.7E-05		2.66E+00	2.6E-03		9.19E+01	1.0E-04		3.46E+00	5.9E-04		2.1E+01
**Cd**	4.0E-03	1.8E-09	2.22E+00	9.8E-04	4.3E-10	5.43E-01	9.8E-04	4.3E-10	5.43E-01	9.8E-04	4.3E-10	5.43E-01	9.8E-04	4.3E-10	5.43E-01	9.8E-04	4.3E-10	5.4E-01
**Sn**	1.7E-03		2.42E+00	3.4E-05		4.74E-02	1.8E-06		2.49E-03	2.2E-04		3.10E-01	6.8E-05		9.41E-02	4.0E-05		5.6E-02
**Sb**	1.5E+00		1.37E+04	2.2E-02		2.00E+02	1.4E-03		1.33E+01	4.1E-02		3.82E+02	3.8E-03		3.55E+01	1.3E-02		1.2E+02
**Ba**	2.6E-03	4.7E-09	5.24E+01	4.9E-04	8.7E-10	9.75E+00	2.1E-04	3.6E-10	4.06E+00	1.4E-03	2.4E-09	2.72E+01	4.4E-04	7.7E-10	8.64E+00	9.6E-04	1.7E-09	1.9E+01
**Hg**	6.4E-04	2.8E-12	1.27E+01	3.1E-03	1.4E-11	6.2E+01	6.4E-04	2.8E-12	1.3E+01	2.9E-03	1.3E-11	5.8E+1	6.4E-04	2.8E-12	1.3E+01	2.3E-04	1.0E-12	4.6E+00
**Pb**																		
**Ta**	1.9E-02		2.66E+01	7.6E-01		1.06E+03	6.9E-01		9.59E+02	2.3E-01		3.20E+02	6.3E-01		8.73E+02	8.1E-01		1.1E+03
**Fe**	4.0E-03		5.61E+00	1.7E-02		2.38E+01	6.0E-03		8.37E+00	1.8E-02		2.51E+01	9.1E-03		1.26E+01	1.4E-02		1.9E+01
**Tl**		7.7E-08			1.3E-07			8.7E-08			8.0E-08			5.2E-08			7.7E-08	
**∑HQ**	1.8E+00	1.2E-07	1.5E+04	8.4E-01	1.7E-07	2.3E+03	7.2E-01	1.0E-07	1.5E+03	3.3E-01	1.2E-07	1.7E+03	6.6E-01	6.8E-08	1.5E+03	8.7E-01	1.1E-07	2.0E+03

Supplementary table 18: Hazard Quotient (HQ) ingestion, inhalation, dermal in soil and dust at various e-waste sites in Ibadan

	Top Soil 0-10cm						Floor dust (control soil)			Direct dust					
**Metals**	**Burning sites**			**Dismantling sites**			**Repair sites**			**Dismantling sites**			**Repair sites**		
	**HQ_{ing}**	**HQ_{inh}**	**HQ_{der}**	**HQ_{ing}**	**HQ_{inh}**	**HQ_{der}**	**HQ_{ing}**	**HQ_{inh}**	**HQ_{der}**	**HQ_{ing}**	**HQ_{inh}**	**HQ_{der}**	**HQ_{ing}**	**HQ_{inh}**	**HQ_{der}**
**V**	4.1E-03	9.1E-10	2.21E+02	4.2E-03	9.4E-10	2.26E+02	5.0E-03	1.1E-09	2.66E+02	7.1E-02	1.6E-08	3.78E+03	8.5E-03	1.9E-09	4.55E+02
**Cr**	1.6E-02	2.1E-09	8.73E+02	1.3E-02	1.8E-09	7.46E+02	1.5E-02	2.0E-09	8.59E+02	1.8E-03	2.4E-10	1.02E+02	1.8E-02	2.4E-09	1.00E+03
**Mn**	2.0E-03	2.5E-08	2.81E+00	1.5E-03	1.9E-08	2.14E+00	2.1E-03	2.6E-08	2.88E+00	4.4E-03	5.4E-08	6.11E+00	2.2E-03	2.7E-08	3.03E+00
**Co**	1.9E-04	4.2E-10	2.66E-01	1.9E-04	4.2E-10	2.66E-01	1.9E-04	4.2E-10	2.66E-01	9.3E-03	2.0E-08	1.28E+01	1.9E-04	4.2E-10	2.66E-01
**Ni**	3.2E-03	1.1E-08	1.13E+02	2.3E-03	7.9E-09	7.87E+01	1.5E-03	5.2E-09	5.20E+01	1.1E-02	3.9E-08	3.87E+02	2.7E-03	9.4E-09	9.44E+01
**Cu**	1.2E-01	1.5E-20	1.70E+02	1.3E-02	1.6E-21	1.79E+01	3.1E-03	3.9E-22	4.30E+00	4.0E-04	5.1E-23	5.59E-01	3.1E-03	3.9E-22	4.27E+00
**Zn**	4.3E-03		5.91E+00	2.3E-03		3.25E+00	1.5E-03		2.05E+00	1.6E-04		2.17E-01	3.2E-03		4.37E+00
**As**	1.3E-01	1.2E-08	5.5E+01	1.3E-02	1.2E-09	5.5+00	3.2E-04	2.8E-11	1.33E-01	1.5E-03	1.4E-10	6.4E-01	6.4E-03	5.6E-10	2.66E+00
**Se**	2.0E-03	2.2E-12	2.77E+00	3.4E-04	3.7E-13	4.69E-01	7.7E-05	8.5E-14	1.07E-01	1.9E-05	2.1E-14	2.66E-02	6.9E-05	7.6E-14	9.59E-02
**Mo**	6.1E-04		8.52E-01	5.5E-04		7.67E-01	4.6E-05		6.39E-02	1.0E-04		1.38E-01	2.1E-04		2.88E-01
**Ag**	1.2E-03		4.26E+01	6.5E-04		2.26E+01	4.2E-04		1.46E+01	1.0E-04		3.59E+00	1.2E-03		3.99E+01
**Cd**	2.7E-03	1.2E-09	1.49E+00	9.8E-04	4.3E-10	5.43E-01	9.8E-04	4.3E-10	5.43E-01	9.8E-04	4.3E-10	5.43E-01	1.0E-03	4.6E-10	5.75E-01
**Sn**	7.8E-04		1.08E+00	7.6E-05		1.05E-01	6.2E-04		8.61E-01	9.6E-07		1.33E-03	3.4E-05		4.70E-02
**Sb**	5.7E-01		5.25E+03	5.0E-02		4.62E+02	1.9E-02		1.78E+02	1.4E-03		1.33E+01	4.6E-02		4.26E+02
**Ba**	1.8E-03	3.2E-09	3.54E+01	1.1E-03	2.0E-09	2.24E+01	9.0E-04	1.6E-09	1.78E+01	1.7E-04	3.0E-10	3.42E+00	2.0E-03	3.6E-09	3.99E+01
**Hg**	5.9E-03	2.6E-11	1.2E+02	1.7E-03	7.3E-12	3.3E+01	9.0E-04	4.0E-12	1.8E+01	6.4E-04	2.8E-12	1.3E+01	3.5E-03	1.5E-11	6.9E+01
**Pb**															
**Ta**	1.9E-02		2.66E+01	5.4E-01		7.46E+02	6.1E-01		8.52E+02	6.9E-01		9.59E+02	1.4E+00		1.92E+03
**Fe**	1.7E-02		2.39E+01	2.7E-02		3.81E+01	2.3E-02		3.24E+01	9.6E-02		1.33E+02	3.1E-02		4.36E+01
**Tl**		1.1E-07			7.9E-08			5.9E-08			9.0E-07			8.4E-08	
**∑HQ**	**9.0E-01**	**1.7E-07**	**6.8E+03**	**6.7E-01**	**1.1E-07**	**2.4E+03**	**6.9E-01**	**9.5E-08**	**2.3E+03**	**8.9E-01**	**1.0E-06**	**5.4E+03**	**1.5E+00**	**1.3E-07**	**4.0E+03**

Supplementary table 19: Hazard Quotient (HQ) ingestion, inhalation, dermal in soil and dust at various e-waste sites and in Aba

	Top Soil 0-10cm						Floor dust						Roadside dust		
**Metals**	**Burning sites**			**Dismantling sites**			**Dismantling sites**			**Repair sites**					
	HQ_{ing}	HQ_{inh}	HQ_{der}	HQ_{ing}	HQ_{inh}	HQ_{der}	HQ_{ing}	HQ_{inh}	HQ_{der}	HQ_{ing}	HQ_{inh}	HQ_{der}	HQ_{ing}	HQ_{inh}	HQ_{der}
**V**	2.2E-03	4.7E-10	1.15E+02	3.5E-03	7.8E-10	1.88E+02	2.0E-03	4.4E-10	1.07E+02	1.3E-03	2.9E-10	6.96E+01	3.2E-03	7.1E-10	1.7E+02
**Cr**	1.3E-02	1.7E-09	7.24E+02	2.5E-02	3.3E-09	1.40E+03	2.4E-02	3.2E-09	1.33E+03	5.4E-03	7.1E-10	2.98E+02	3.8E-03	5.1E-10	2.1E+02
**Mn**	1.1E-03	1.4E-08	1.59E+00	1.5E-03	1.8E-08	2.05E+00	1.8E-03	2.2E-08	2.49E+00	8.4E-04	1.0E-08	1.17E+00	7.5E-04	9.3E-09	1.0E+00
**Co**	1.9E-04	4.2E-10	2.66E-01	1.9E-04	4.2E-10	2.66E-01	1.9E-04	4.2E-10	2.66E-01	1.9E-04	4.2E-10	2.66E-01	1.9E-04	4.2E-10	2.7E-01
**Ni**	3.0E-03	1.0E-08	1.04E+02	5.3E-03	1.9E-08	1.85E+02	5.2E-03	1.8E-08	1.80E+02	8.0E-04	2.8E-09	2.78E+01	5.6E-04	1.9E-09	1.9E+01
**Cu**	4.3E-02	5.4E-21	5.90E+01	7.6E-02	9.5E-21	1.05E+02	1.3E-01	1.6E-20	1.81E+02	1.8E-03	2.3E-22	2.54E+00	2.2E-04	2.8E-23	3.1E-01
**Zn**	4.5E-03		6.31E+00	7.2E-03	#DIV/0!	1.00E+01	6.9E-03		9.59E+00	6.1E-04		8.41E-01	1.5E-04		2.1E-01
**As**	3.1E-02	2.7E-09	1.28E+01	5.1E-02	4.5E-09	2.13E+01	9.1E-02	8.0E-09	3.78E+01	3.2E-04	2.8E-11	1.33E-01	3.2E-04	2.8E-11	1.3E-01
**Se**	1.9E-04	2.1E-13	2.66E-01	8.8E-04	9.7E-13	1.22E+00	3.1E-04	3.4E-13	4.26E-01	1.9E-05	2.1E-14	2.66E-02	1.9E-05	2.1E-14	2.7E-02
**Mo**	1.1E-03		1.49E+00	1.3E-03		1.81E+00	1.3E-03		1.86E+00	3.8E-05		5.33E-02	3.8E-05		5.3E-02
**Ag**	6.1E-04		2.13E+01	1.1E-03		3.86E+01	3.1E-03		1.07E+02	7.7E-05		2.66E+00	7.7E-05		2.7E+00
**Cd**	1.9E-04	8.5E-11	1.07E-01	9.2E-03	4.1E-09	5.11E+00	1.2E-02	5.1E-09	6.39E+00	9.8E-04	4.3E-10	5.43E-01	9.8E-04	4.3E-10	5.4E-01
**Sn**	5.2E-05		7.19E-02	1.5E-04		2.11E-01	4.7E-04		6.57E-01	2.2E-04		3.00E-01	3.5E-06		4.9E-03
**Sb**	4.7E-02		4.35E+02	1.9E-01		1.80E+03	3.7E-01		3.39E+03	9.6E-03		8.88E+01	1.1E-03		9.8E+00
**Ba**	1.9E-03	3.3E-09	3.70E+01	7.5E-03	1.3E-08	1.49E+02	1.9E-03	3.4E-09	3.77E+01	4.1E-04	7.3E-10	8.18E+00	3.3E-04	5.8E-10	6.5E+00
**Hg**	6.4E-04	2.8E-12	1.3E+01	6.4E-04	2.8E-12	1.3E+01	6.4E-04	2.8E-12	1.3E+01	6.4E-04	2.8E-12	1.3E+01	1.3E+01	5.6E-12	2.5E+01
**Pb**															
**Ta**	1.9E-02		2.66E+01	3.8E-02		5.33E+01	3.8E-02		5.33E+01	6.5E-01		9.05E+02	1.0E+00		1.4E+03
**Fe**	2.8E-02		3.91E+01	3.8E-02		5.33E+01	5.3E-02		7.40E+01	1.0E-02		1.43E+01	9.8E-03		1.4E+01
**Tl**		9.7E-08			4.4E-08			8.4E-08			6.5E-08			9.3E-08	
**∑HQ**	**2.0E-01**	**1.3E-07**	**1.6E+03**	**4.6E-01**	**1.1E-07**	**4.0E+03**	**7.4E-01**	**1.4E-07**	**5.5E+03**	**6.9E-01**	**8.1E-08**	**1.4E+03**	**1.0E+00**	**1.1E-07**	**1.8E+03**

Supplementary table 20: Oral Reference Dose(RfD), Inhalation Reference dose(RfC), gastrointestinal absorption factor (GIABS) for metals

Metals	RfD	RfC	GIABS
**V**	5.00E-03	1.00E-04	2.60E-02
**Cr**	3.00E-03	1.00E-04	2.50E-02
**Mn**	1.40E-01	5.00E-05	1.00E+00
**Co**	3.00E-03	6.00E-06	1.00E+00
**Ni**	1.10E-02	1.40E-05	4.00E-02
**Cu**	4.00E-02	1.40E+09	1.00E+00
**Zn**	3.00E-01		1.00E+00
**As**	3.00E-04	1.50E-05	1.00E+00
**Se**	5.00E-03	2.00E-02	1.00E+00
**Mo**	5.00E-03		1.00E+00
**Ag**	5.00E-03		4.00E-02
**Cd**	1.00E-03	1.00E-05	2.50E-02
**Sn**	6.00E-01		1
**Sb**	4.00E-04	0.00E+00	1.50E-01
**Ba**	2.00E-01	5.00E-04	7.00E-02
**Hg**	3.00E-04	3.00E-04	0.07
**Pb**	8.50E-03	1.20E-05	
**Ta**	1.00E-05		1.00E+00
**Fe**	7.00E-01		1.00E+00
**Ti**		1.00E-04	1

Supplementary table21: Cancer risk of BDE-209 through ingestion, inhalation, dermal in soil and dust at various e-waste sites and in Lagos

PBDE	SF	Top soil									Floor dust (control soil)						Roadside dust		
		Burning sites			Dismantling sites			Repair sites			Dismantling sites			Repair sites			Dismantling sites		
		R_{ing}	R_{inh}	R_{der}	R_{ing}	R_{inh}	R_{der}	R_{ing}	R_{inh}	R_{der}	R_{ing}	R_{inh}	R_{der}	R_{ing}	R_{inh}	R_{der}	R_{ing}	R_{inh}	R_{der}
BDE-209	7.0E-04	4.73E-09		6.6E-06	4.0E-10		5.6E-07	2.3E-13		3.2E-10	8.1E-10		1.1E-06	1.2E-10		1.6E-07	2.6E-10		3.6E-07

Supplementary table 22: Cancer risk of BDE-209 through ingestion, inhalation, dermal in soil and dust at various e-waste In Ibadan

PBDE	SF	Top soil						Floor dust (control soil)			Direct dust					
		Burning sites			Dismantling sites			Repair sites			Dismantling sites			Repair sites		
		R_{ing}	R_{inh}	R_{der}	R_{ing}	R_{inh}	R_{der}	R_{ing}	R_{inh}	R_{der}	R_{ing}	R_{inh}	R_{der}	R_{ing}	R_{inh}	R_{der}
BDE-209	7.0E-04	1.6E-06		2.2E-03	3.5E-09	1.5E-17	4.8E-06	2.2E-06	9.5E-15	3.0E-03	8.5E-06	3.8E-14	1.2E-02	3.9E-07	1.7E-15	5.5E-04

Supplementary table 23: Cancer risk of BDE-209 throughingestion, inhalation, dermal in soil and dust at various e-waste sites and in Aba

PBDE	SF	Top soil			Floor dust (control soil)			Roadside dust		
		Burning sites			Repair sites			Repair sites		
		R_{ing}	R_{inh}	R_{der}	R_{ing}	R_{inh}	R_{der}	R_{ing}	R_{inh}	R_{der}
BDE-209	7.0E-04	2.8E-08		3.9E-05	1.4E-07	6.3E-16	2.05-04	1.9E-08	8.2E-17	2.6E-05

Supplementary table 24: Cancer risk of metals through ingestion, inhalation, dermal in soil and dust at various e-waste sites and in Lagos

Metals				Top soil									Floor dust (control soil)						Roadside dust		
				Burning sites			Dismantling sites			Repair sites			Dismantling sites			Repair sites			Dismantling sites		
	Sfora	IUR	GIABS	R_{ing}	R_{inh}	R_{der}	R_{ing}	R_{inh}	R_{der} =	R_{ing} =	R_{inh}	R_{der}	R_{ing}	R_{inh}	R_{der}	R_{ing}	R_{inh}	R_{der}	R_{ing}	R_{inh}	R_{der}
Cr	5.0E-01	8.4E-02	2.5E-02	1.3E-05	9.8E-15	4.6E-04	2.0E-05	1.5E-14	6.9E-04	9.5E-06	7.0E-15	3.3E-04	1.5E-05	1.1E-14	5.2E-04	1.1E-05	8.1E-15	3.8E-04	1.2E-05	8.9E-15	4.2E-04
Co		9.0E-03	1	0	2.3E-17			3.9E-17			2.3E-17			2.3E-17			2.3E-17			4.3E-17	
Ni		2.6E-04	4.0E-02	0	8.6E-17			2.2E-17			4.4E-17			4.4E-17			1.2E-17			3.7E-17	
As	1.5	4.3E-03	1	4.8E-05	6.1E-16	2.0E-02	2.8E-06	3.6E-17	1.2E-03	2.1E-06	2.6E-17	8.6E-04	3.3E-06	4.2E-17	1.4E-03	1.4E-07	1.8E-18	6.0E-05	7.5E-06	9.5E-17	3.1E-03
Cd		1.8E-03	2.5E-02	0	3.2E-17			7.8E-18			7.8E-18			7.8E-18			7.8E-18			7.8E-18	
Hg		3.00E-04	0.07	0	2.5E-19			1.2E-18			2.5E-19			1.2E-18			2.5E-19			9.1E-20	
Pb	8.50E-03	1.20E-05	1	2.1E-05	1.3E-16	2.9E-02	6.5E-07	4.0E-18	9.0E-04	4.0E-08	2.5E-19	5.5E-05	9.0E-07	5.6E-18	1.3E-03	1.9E-07	1.2E-18	2.6E-04	1.2E-06	7.6E-18	1.7E-03
**HI**				8.2E-05	1.1E-14	4.9E-02	2.3E-05	1.5E-14	2.8E-03	1.2E-05	7.1E-15	1.2E-03	1.9E-05	1.1E-14	3.1E-03	1.1E-05	8.2E-15	7.0E-04	2.1E-05	9.1E-15	5.2E-03

IUR = Inhalation Unit Risk. SF= Slope factor. GIABS = gastrointestinal absorption factor

Supplementary table 25: Cancer risk of metals through ingestion, inhalation, dermal in soil and dust at various e-waste sites and in Ibadan

Metals	Sforal	IUR	GIABS	Top soil						Floor dust (control soil)						Direct dust		
				Burning sites			Dismantling sites			Dismantling sites			Dismantling sites			Repair sites		
				$R_{ing =}$	R_{inh}	R_{derl}	$R_{ing =}$	R_{inh}	R_{der}	R_{ing}	R_{inh}	R_{der}	R_{ing}	R_{inh}	$R_{der =}$	R_{ing}	R_{inh}	R_{der}
**Cr**	5.0E-01	8.4E-02	2.5E-02	2.4E-05	1.8E-14	8.2E-04	2.0E-05	1.5E-14	7.0E-04	2.3E-05	1.7E-14	8.1E-04	2.7E-06	2.0E-15	9.5E-05	2.7E-05	2.0E-14	9.4E-04
**Co**		9.0E-03	1		2.3E-17			2.3E-17			2.3E-17			1.1E-15			2.3E-17	
**Ni**		2.6E-04	4.0E-02		4.1E-17			2.9E-17			1.9E-17			1.4E-16			3.4E-17	
**As**	1.5	4.3E-03	1	5.9E-05	7.5E-16	2.5E-02	5.9E-06	7.5E-17	2.5E-03	1.4E-07	1.8E-18	6.0E-05	6.9E-07	8.7E-18	2.9E-04	2.9E-06	3.6E-17	1.2E-03
**Cd**		1.8E-03	2.5E-02		2.1E-17			7.8E-18			7.8E-18			7.8E-18			8.2E-18	
**Hg**		3.00E-04	0.07		2.3E-18			6.6E-19			3.6E-19			2.5E-19			1.4E-18	
**Pb**	8.50E-03	1.20E-05	1	7E-05	4.4E-16	9.7E-02	8.0E-06	5.0E-17	1.1E-02	3.1E-06	1.9E-17	4.3E-03	1.6E-09	1.0E-09	2.3E-06	1.5E-06	9.2E-18	2.0E-03
**∑HQ=HI**				**1.5E-04**	**1.9E-14**	**1.2E-01**	**3.4E-05**	**1.5E-14**	**1.4E-02**	**2.6E-05**	**1.7E-14**	**5.2E-03**	**3.4E-06**	**3.3E-15**	**3.9E-04**	**3.1E-05**	**2.0E-14**	**4.2E-03**

Supplementary table 26: Cancer risk of metals through ingestion, inhalation, dermal in soil and dust at various e-waste sites and in Aba

				Top soil						Floor dust (control soil)						Roadside dust		
				Burning sites			Dismantling sites			Dismantling sites			Repair sites			Repair sites		
**Metals**	**Sforal**	**IUR**	**GIABS**	R_{ing}	R_{inh}	R_{der}	R_{ing}	R_{inh}	R_{der}	R_{ing}	R_{inh}	R_{der}	R_{ing}	R_{inh}	$R_{der =}$	$R_{ing =}$	$R_{inh =}$	$R_{der =}$
**Cr**	5.0E-01	8.4E-02	2.5E-02	2.0E-05	1.5E-14	6.8E-04	3.8E-05	2.8E-14	1.3E-03	3.6E-05	2.7E-14	1.3E-03	8.1E-06	6.0E-15	2.8E-04	1.9E-03	4.3E-15	2.7E+00
**Co**		9.0E-03	1	0	2.3E-17			2.3E-17			2.3E-17			2.3E-17			2.3E-17	
**Ni**		2.6E-04	4.0E-02	0	3.8E-17			6.7E-17			6.6E-17			1.0E-17			7.0E-18	
**As**	1.5	4.3E-03	1	1.4E-05	1.7E-16	5.8E-03	2.3E-05	2.9E-16	9.6E-03	4.1E-05	5.2E-16	1.7E-02	1.4E-07	1.8E-18	6.0E-05	4.8E-04	1.8E-18	2.0E-01
**Cd**		1.8E-03	2.5E-02	0	1.5E-18			7.3E-17			9.1E-17			7.8E-18			7.8E-18	
**Hg**		3.00E-04	0.07	0	2.5E-19			2.5E-19			2.5E-19			2.5E-19			5.1E-19	
**Pb**	8.50E-03	1.20E-05	1	4.9E-06	3.1E-17	6.8E-03	7.4E-06	4.6E-06	1.0E-02	1.2E-05	7.7E-17	1.7E-02	4.9E-07	3.0E-18	6.8E-04	1.1E-07	6.9E-19	1.5E-04
**∑HQ=HI**				**3.8E-05**	**1.5E-14**	**1.3E-02**	**6.8E-05**	**2.9E-14**	**2.1E-02**	**8.9E-05**	**2.8E-14**	**3.5E-02**	**8.7E-06**	**6.0E-15**	**1.0E-03**	**6.0E-06**	**4.3E-15**	**4.1E-04**

Supplementary table 27: Total HI Estimate for PBDEs and Metals for Cancer Risks (log transformed data)

Samples	Location	Activity	Ingestion	Inhalation	Dermal contact
Direct dust	Ibadan	Dismantling	-5.47	-14.52	-3.41
		Repair	-4.51	-13.7	-2.38
Floor dust	Lagos	Dismantling	-4.72	-13.96	-2.51
		Repair	-4.96	-14.1	-3.15
	Ibadan	Repair	-4.58	-13.77	-2.28
	Aba	Dismantling	-4.05	-13.55	-1.46
		Repair	-5.06	-14.22	-3
Roadside Dust	Lagos	Dismantling	-4.68	-14.05	-2.28
	Aba	Repair	-5.22	-14.4	-3.39
Soil	Lagos	Burning	-4.09	-13.96	-1.31
		Dismantling	-4.64	-13.82	-2.55
		Repair	-4.92	-14.15	-2.92
	Ibadan	Burning	-3.82	-13.72	-0.92
		Dismantling	-4.47	-13.82	-1.85
	Aba	Burning	-4.42	-13.82	-1.89
		Dismantling	-4.17	-13.54	-1.68
Safe Limit			-6	-6	-6

Supplementary table 28: Cumulative HI Estimate for PBDEs and Metals for non-cancer effects and Cancer Risks (log transformed data)

Samples	Location	Activity	Non-cancer Effects	Cancer Risks
Direct dust	Ibadan	Dismantling	3.73	-3.41
		Repair	3.6	-2.38
Floor dust	Lagos	Dismantling	3.23	-2.51
		Repair	3.18	-3.15
	Ibadan	Repair	3.36	-2.28
	Aba	Dismantling	3.74	-1.46
		Repair	3.15	-3
Roadside Dust	Lagos	Dismantling	3.3	-2.28
	Aba	Repair	3.26	-3.39
Soil	Lagos	Burning	4.18	-1.31
		Dismantling	3.36	-2.55
		Repair	3.18	-2.92
	Ibadan	Burning	3.83	-0.92
		Dismantling	3.38	-1.85
	Aba	Burning	3.2	-1.89
		Dismantling	3.6	-1.68
Safe Limit			0	-6

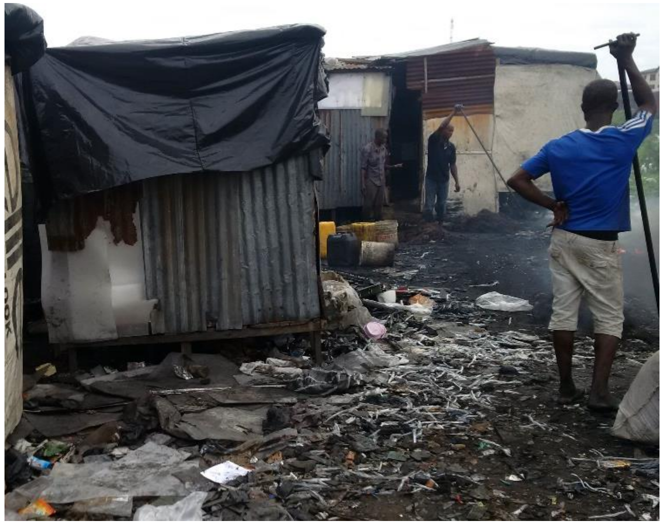

Supplementary Fig. 1D

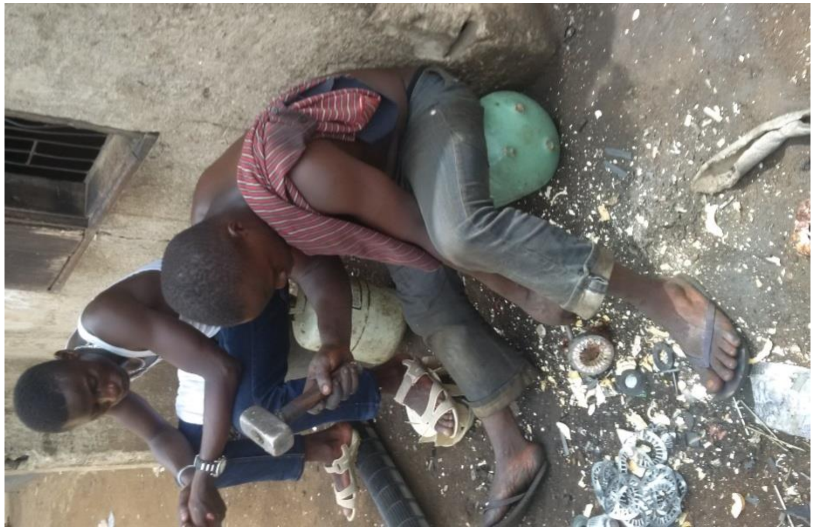

Supplementary Fig. 1E

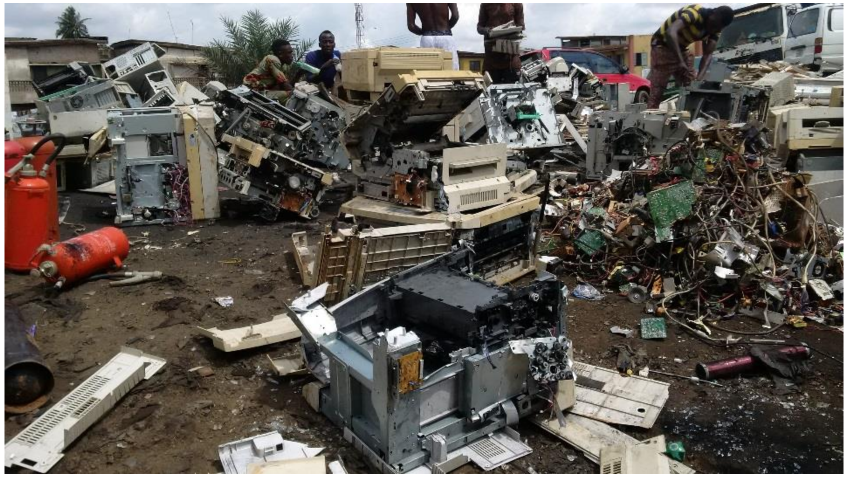

Supplementary Fig. 1F

Supplementary Fig. 1D-F: Photos of e-waste workers at the e-waste recycling sites showing no use of PPE
